# Yield impact of source-sink-regulated senescence in hybrid maize and genetic architecture in exPVP inbreds

**DOI:** 10.1007/s00122-026-05210-z

**Published:** 2026-04-27

**Authors:** Mark Gee, Rajdeep S. Khangura, Mitchell R. Tuinstra

**Affiliations:** 1 Department of Agronomy, Purdue University, 915 Mitch Daniels Blvd, West Lafayette, IN 47907 USA; 2 Department of Plant and Agroecosystem Sciences, University of Wisconsin-Madison, 1575 Linden Dr, Madison, WI 53706 USA

## Abstract

Prior genetic studies on source-sink-regulated senescence in maize have characterized some genes associated with the trait, but the impact on yield is unknown and the molecular understanding of this trait is still in its infancy. We phenotyped source-sink-regulated senescence in a commercial maize exPVP inbred population and assessed its correlation with yield and other commercially relevant agronomic traits in testcrosses under field conditions. Genome-wide association studies were conducted in both inbred and F1 hybrid populations. The effect of most significant alleles for senescence regulation in inbred germplasm was compared with their effect on yield in the F1 hybrids to investigate genetic co-regulation. Source-sink-regulated senescence of inbred parents was correlated with the yield of intra-family hybrids but was not generally correlated with the yield of hybrids made from crosses between two heterotic groups. Stronger senescence response was positively correlated with increased kernel numbers. Several genetic loci were identified as highly significant, including loci on chromosomes 1 and 4, which overlap kernel trait hotspots from previous studies. Single factor testing of DELLA mutants supports the conclusion that the GA-DELLA signaling pathway attenuates the senescence response to sink disruption in maize. The genetic basis of source-sink-regulated senescence may have contributed to evolutionary fitness in inbred lines through increased kernel numbers but are not strong drivers of yield in the modern F1 hybrids. This could permit selection of loci regulating senescence for inbred improvement without affecting yield in commercial hybrid breeding.

**Supplementary Information:**

The online version contains supplementary material available at 10.1007/s00122-026-05210-z.

## Introduction

Alteration of late-season senescence in maize has led to significant commercial yield increases. Natural senescence is complex and regulated by several biological processes including chlorophyll catabolism, sugar signaling, and photoperiod response (Lim et al. 2007; Wingler et al. 2009; Sekhon et al. 2019; Domínguez and Cejudo 2021). Uncovering the genetic and physiological mechanisms controlling the different forms of senescence will give us a deeper understanding of this fundamental process needed to drive future yield increases.

Senescence can be triggered by the integration of many biochemical signals, and the mechanisms which feed into senescence signaling pathways are an active area of investigation. A distinction has been made between senescence in the presence of high sugar and low sugar levels (Wingler et al. 2009). High-sugar senescence is exacerbated by low nitrogen availability, which corresponds to grain filling conditions where senescence confers an evolutionary advantage. In this scenario, sufficient sugar production but insufficient nitrogen can be counteracted by breaking down photosynthetic machinery and remobilizing the nitrogen to sink tissues. Low-sugar senescence corresponds to adverse conditions in which leaves do not produce enough carbohydrates to justify their maintenance costs. Dark-induced senescence is a faster acting trigger that achieves the same effect of low-sugar senescence by preventing the plant from wasting resources on leaves that do not produce sufficient resources to justify their existence. A transcriptomic analysis suggested that developmental leaf senescence is more similar to leaf punches undergoing high-sugar/low-N senescence than dark-induced senescence (Wingler et al. 2009).

Sink disruption by either pollination prevention or ear removal is a simple method to induce high sugar levels and study the senescence response to source-sink balance in isolation from other senescence triggers. Physiological studies of sink removal in the second half of the twentieth century found contrasting senescence responses to sink removal in maize, but the most common response was an increase in sugar accumulation followed by red pigmentation and acceleration of senescence (Allison and Weinmann 1970; Barnett and Pearce 1983; Borrás et al. 2003; Christensen et al. 1981; Crafts-Brandner et al. 1984b; Moss 1962; Rajcan and Tollenaar 1999a; Tollenaar and Daynard 1982). This work spurred deeper investigation into the regulation of maize senescence by source-sink balance and led to the discovery of a complex, genotypic response to source-sink disruption.

Modern investigation into source-sink-regulated senescence in maize has sought to uncover the genetic and biochemical mechanisms underlying the diversity of the phenotype. In B73, visual onset of source-sink-regulated senescence symptoms occurs approximately 24 days after anthesis (DAA) but increases in free glucose and starch, and loss of chlorophyll can be analytically detected in leaves twelve days after anthesis in sink-disrupted plants (Sekhon et al. 2012). A study of 31 inbred lines from Midwest germplasm found evidence of a source-sink senescence response across heterotic groups and a potential link to late-season staygreen (Kumar et al. 2019). Lines which were insensitive to sink disruption partitioned excess photosynthate to alternative sink tissues and nonstructural carbohydrates. Partitioning of nonstructural carbohydrates to the internodes at and above the primary ear‐bearing node was highly correlated with reduced senescence response while partitioning of carbohydrates to the cob was least correlated. Time‐course analyses of sensitive (B73) and resistant (PHG35) inbred lines showed differential sugar partitioning and regulation (Kumar et al. 2019). In response to sink disruption, PHG35 upregulated key sugar transporters, resulting in decreased sugar levels in leaves and increased sugar levels in internodes. Elevated hemicellulose quantities in PHG35 internodes indicated the cell wall as a significant alternative sink. Increased accumulation of trehalose‐6‐phosphate and decreased transcript levels of snf1‐related protein kinase1 in B73 non-pollinated plants provide further evidence of sugar signaling differences. However, transcriptional analysis of the SWEET family of sugar transporters uncovered a complicated response of both up- and downregulation of transporters. Thousands of differentially expressed genes were identified, and the data were not able to narrow the list of candidates. Consistent with this work, another study in maize using a semidominant mutant of a chlorophyll biosynthetic enzyme *oil yellow1 (oy1)* with reduced photosynthetic rate and reduced levels of free sugars and starch found that the mutant exhibited suppressed senescence after sink disruption (Khangura et al. 2020). Using *oy1* mutant and its modifier in an isogenic B73 background, Khangura et al. (2020) also demonstrated that mutants with a severe reduction in chlorophyll displayed delayed senescence compared to mutants with suppressed chlorosis.

Kumar et al. (2023) identified maize lines that were sensitive to sink removal and showed increased levels of starch at 7 DAA and increased levels of glucose and sucrose at 15 DAA. A subset of insensitive maize lines did not manifest differences until 30 DAA. Hormone signaling was also affected with hexokinase levels in sensitive genotypes increasing in response to sink disruption at 21 DAA. In both sensitive and non-sensitive genotypes, cytokinin levels increased with age in pollinated plants but did not increase in non-pollinated plants. Biparental mapping using intermated B73xMo17 recombinant inbred lines revealed 12 QTLs for senescence response to sink disruption which were narrowed via analysis of near-isogenic lines. B73 is highly sensitive to sink-disruption-induced senescence, whereas Mo17 is less sensitive than B73. The comparison of transcriptional responses in B73 and Mo17 in response to non-pollination found 10,331 differentially expressed genes in B73 and only 2178 genes in Mo17. Increase in reactive oxygen species was identified as one of the earliest responses to non-pollination followed by the onset of endoplasmic reticulum stress. ABA was identified as a potential intermediate in the ROS and ER stress response. The Wisconsin diversity panel was used in the same study to investigate natural diversity of the source-sink senescence phenotype and identified 24 significant SNP through GWA. Using a combination of linkage analysis, transcriptional data, and gene co-expression networks, a list of 24 candidate genes were identified by Kumar et al. (2023). One of the significant SNPs on chromosome 7 was supported by all criteria and identified *ccp4* (Zm0001d021615) that encodes a cathepsin B cysteine protease as a *bona fide* regulator of sugar-mediated senescence.

Despite the significant work to understand the genetic and biochemical mechanisms of source-sink-regulated senescence, limited research has explored its implications on commercially relevant traits. A study of commercial maize hybrids investigated the relationship between source-sink-regulated senescence and leaf staygreen and found weak correlation (Willman et al. 1987). However, no study in maize has explored correlation of source-sink-regulated senescence with yield. We explored this relationship by correlating the source-sink-regulated senescence phenotypes of a panel of exPVP inbreds with yield and other agronomic traits of their derived testcross hybrids. The comparison of overall genotype effect showed weak correlation in specific heterotic pools, suggesting a limited role for selection on source-sink-regulated senescence to improve hybrid yields. Our approach to characterize senescence and yield using commercially relevant inbred and testcross hybrid populations can be broadly applied to test the relationship between other physiological and agronomic traits.

## Materials and methods

### Plant materials, experimental design, and exPVP phenotyping

A population of 343 exPVP inbred lines were selected to represent the diversity of commercial germplasm that has recently moved into the public domain (Beckett et al. 2017). Senescence phenotypes of these inbred lines were evaluated during the summer of 2022 and 2023 (West Lafayette, IN) with two replicates each year using a randomized complete block design. To quantify the source-sink-regulated senescence response and separate it from baseline chlorophyll variation and other senescence mechanisms, we measured the senescence of both pollinated and non-pollinated plants within the same plot. Within each plot, five plants received a non-pollination treatment through a combination of covering ears before silk emergence and ear removal in the case of silk exposure. Previous work has shown that both methods of sink disruption are acceptable for inducing the source-sink-regulated senescence response (Renaud 2015). The rest of the plants in the plot were unaltered and left to open pollinate. Three representative plants for each pollination treatment were measured based on uniformity relative to the plot. To quantify progression of senescence, non-destructive chlorophyll content index (CCI) was measured using a chlorophyll content meter model CCM-200 plus (Opti-Sciences, Inc., Hudson, NH). To ensure a uniform growth stage across genotypes, CCI was measured at 425 growing degree days (GDD) and 600 GDD (± 50GDD) after the plot had reached 50% anthesis. The 600 GGDC measurement was only taken during the 2023 season. On individual plants, measurements were taken on the leaf above the primary ear, half-way between the leaf base and tip, and half-way between the midrib and leaf edge. Before collecting measurements, ears were inspected to ensure pollination or non-pollination according to the experimental design. Plants that had completely senesced and dried were assigned a CCI value of zero. Plot CCI values were calculated as the mean of three plants for each pollination treatment. To ensure high-quality data, measurements were removed from the analysis if they fell outside the acceptable GDD range, if all the pollinated plants in a plot had completely senesced at the time of measurement, if disease symptoms initiated unusual senescence, if less than two plants per treatment could be measured, or if measurements were identified as an outlier using the statistical analyses described in this paper.

### Quantification of source-sink-regulated senescence

Previous literature has reported numerous methods for quantifying the senescence response to sink disruption and separating the effect from baseline chlorophyll levels and other senescence mechanisms. Most of these methods are based on measuring the senescence of non-pollinated plants and adjusting the value based on the measurement of pollinated plants from the same genotype. We compared these methods and determined their usefulness based on heritability and correlation with other traits. Willman reported the rate of senescence induced by ear removal based on a visual score of senescence at 30 and 44 DAA (Willman et al. 1987). Kumar defined source-sink-regulated senescence as the difference in photosynthetic rates between pollinated and non-pollinated plants normalized by the pollinated photosynthetic rate to account for differing baseline chlorophyll levels across genotypes and called this phenotype SSRS (Kumar et al. 2019). We use the term SSRS in the remainder of this paper to refer specifically to the phenotype defined by Kumar and use the term “source-sink regulated senescence” to refer to the biological phenomena. Kumar later quantified the senescence trait using the photosynthetic rate of non-pollinated plants alone and also used the difference in photosynthetic rates between pollinated and non-pollinated plants (Kumar et al. 2023). With these examples as precedent, we used CCI data collected at two timepoints to derive multiple phenotypes described below for quantifying the senescence response to sink disruption.

$$\begin{aligned} & {\mathrm{CCI}} = {\text{Value of CCI for a given pollination}} \\ & {\text{treatment at a given GDD accumulation}} \\ \end{aligned}$$

$${\text{Difference in CCI}} = {\text{CCI Pollinated}} - {\text{CCI Non Pollinated}}$$

$${\text{SSRS = }}\frac{{{\text{CCI Pollinated}} - {\text{CCI Non Pollinated}}}}{{\text{CCI Pollinated}}}$$

$${\text{Rate of Senescence = }}\left( {\frac{{{\mathrm{CCI}}@{\mathrm{Time2}} - {\mathrm{CCI}}@{\mathrm{Time1}}}}{{{\mathrm{GDD}}@{\mathrm{Time2}} - {\mathrm{GDD}}@{\mathrm{Time1}}}}} \right)$$

$${\text{Difference in Rate}} = {\text{Rate Non Pollinated}} - {\text{Rate Pollinated}}$$

Traits were calculated on a plot basis and best linear unbiased predictors (BLUPs) were estimated using a mixed-linear model described below that controlled for spatial variation by including fixed effects for block, row, and range. Variables were nested within year to account for the fact that soil–trait interactions and genetic–trait interactions vary between seasons. Models were fit, and the variance estimates were calculated using restricted maximum likelihood (REML) implemented in the R package lme4 (Bates et al. 2015).

$$\begin{aligned} {\text{Trait }}\sim { } & {\mathrm{mean}} + \left( {\mathrm{1|Genotype}} \right) + \left( {\mathrm{1|Year}} \right) + ({\mathrm{1|Year:Genotype}}) \\ + ({\mathrm{1|Year:Block}}) + ({\mathrm{1|Year:Row}}) + ({\mathrm{1|Year:Range}}) \\ \end{aligned}$$

An additional trait, CCI non-pollinated adjusted, was defined to control for the baseline chlorophyll variation across genotypes by including the pollinated CCI as a fixed effect according to the formula below. This method is commonly used in plant and animal breeding (Garrick 2010) and is expected to control for the impact of baseline levels of chlorophyll with less bias than the normalization method of SSRS.

$$\begin{aligned} & {\mathrm{CCI}} {\text{Non Pollinated adjusted}} \sim {\mathrm{mean}} + \left( {\mathrm{1|Genotype}} \right) \\ & \quad + \left( {\mathrm{1|Year}} \right) + ({\text{1|Year:Genotype) }}\left( {\mathrm{1|Year:Rep}} \right) \\ & \quad + \left( {\mathrm{1|Year:Row}} \right) + \left( {\mathrm{1|Year:Range}} \right) + {\mathrm{CCI}} {\mathrm{Pollinated}} \\ \end{aligned}$$

Broad-sense heritability was calculated for all traits on an entry-mean basis (Piepho and Möhring 2007) using variance components estimated through REML.

$$H^{2} = \frac{{\sigma_{G}^{2} }}{{\sigma_{G}^{2} + \frac{{\sigma_{{{\mathrm{GY}}}}^{2} }}{Y} + \frac{{\sigma_{ \in }^{2} }}{{Y * {\mathrm{rep}}}}}}$$

Traits at 600 GDD and rate of senescence could only be estimated for 2023 because that is the only year when the 600 GDD data were collected. Therefore, year-and-year interaction terms were not included when estimating BLUP and heritability is expected to be higher.

Significance of model terms were estimated through REML-likelihood ratio tests with single term deletions of random effects using the R package lmerTest (Kuznetsova et al. 2017). The heritability of traits is reported in Table 1, and significance of model terms is reported in Supplemental Table S1. Genotype and genotype-by-year interactions were significant in all cases. Range, row, and replication were not always significant but were retained in the model for consistency with the experimental design.

**Table 1:** Trait descriptions and entry mean heritability. Only one year of data was collected for exPVP 600GDD measurements so the heritability of these measurements and rates of change are higher than would be observed in a multi-year trial

Trait category	Traits	Units	Heritability	Definition
exPVP inbreds at 425 GDD	Pol	CCI	0.72	Chlorophyll content index of pollinated plants
	NonPol	CCI	0.69	Chlorophyll content index of non-pollinated plants
	Difference	CCI	0.56	Difference in chlorophyll content index between pollinated and non-pollinated plants
	SSRS	CCI CCI-1	0.62	Difference in chlorophyll content index divided by the chlorophyll content index of pollinated plants
	NonPoladj	CCI	0.61	Chlorophyll content index of non-pollinated plants adjusted for the fixed effect of Pol
exPVP inbreds at 600 GDD	Pol	CCI	0.75	Chlorophyll content index of pollinated plants
	NonPol	CCI	0.82	Chlorophyll content index of non-pollinated plants
	Difference	CCI	0.80	Difference in chlorophyll content index between pollinated and non-pollinated plants
	SSRS	CCI CCI-1	0.81	Difference in chlorophyll content index divided by the chlorophyll content index of pollinated plants
	NonPoladj	CCI	0.82	Chlorophyll content index of non-pollinated plants adjusted for the fixed effect of Pol
exPVP rate of change from 425 to 600 GDD	Pol	CCI GDD-1	0.28	Chlorophyll content index of pollinated plants
	NonPol	CCI GDD-1	0.67	Chlorophyll content index of non-pollinated plants
	Difference	CCI GDD-1	0.59	Difference in chlorophyll content index between pollinated and non-pollinated plants
	SSRS	CCI CCI-1 GDD-1	0.60	Difference in chlorophyll content index divided by the chlorophyll content index of pollinated plants
	NonPoladj	CCI GDD-1	0.67	Chlorophyll content index of non-pollinated plants adjusted for the fixed effect of Pol
Hybrid testcross	Yield	bu acre-1	0.82	Yield of Plots at 15% moisture
	NDVI Senescence	NDVI NDVI-1	0.79	Change in NDVI from anthesis to 550 GDD post-anthesis divided by NDVI at anthesis
Hybrid testcross ear pssshotometry	PHTKPE	count ear − 1	0.53	Total number of kernels per ear
	PHTKPR	count ear − 1	0.61	Total number of kernels per row
	PHTKR	count ear − 1	0.55	Kernel row number
	KERFIL	cm2 (cm2) − 1	0.54	Percent of total ear area with filled kernels
	TKERAB	cm cm − 1	0.49	Percent of ear length affected by kernel abortion
	SCTTER	cm2(cm2) − 1	0.54	Percent of ear area lost due to scatter grain
	EARLGT	cm ear − 1	0.66	Total length of cob
	EARWTH	cm ear − 1	0.71	Width of ear including kernels and cob
	EARCW	–	0.69	Ear central width
	EARAREA	cm2 ear − 1	0.59	Ear area
	EARVOL	cm2 ear − 1	0.56	Ear volume
	EARPER	cm ear − 1	0.68	Ear perimeter
	EARRC	count ear − 1	0.60	Ring count
	EARTR	cm2 ear − 1	0.64	Ear tip ratio
	KERCC	–	0.49	Kernel central count
	KERARE	cm2 kernel − 1	0.63	Average area per kernel
	KERYLD_g	g kernel-1	0.50	Average single kernel mass
	KERLEN	cm kernel − 1	0.64	Average kernel length
	KERWTH	cm kernel − 1	0.60	Average kernel width
	KERMEAND	cm kernel − 1	0.66	Kernel mean diameter
	KERPER	cm kernel − 1	0.66	Kernel perimeter

### Testcross evaluation

In a previous study, F1 hybrid testcrosses were made between 200 of the exPVP inbred lines and two testers, 2FACC and PHP02, and evaluated as published (Tolley 2023). In brief, the F1 hybrids were planted during the 2021 and 2022 seasons in four-row plots at a population of 74,000 seeds ha^−1^ on May 23, 2021, and May 12, 2022, in a randomized complete block design with two replications. Grain yields were obtained from two inner plot rows of each four-row plot on September 30, 2021, and October 3, 2022, using a Kincaid plot combine and were adjusted to 15% moisture. Hyperspectral data were collected at regular intervals throughout the growing season, and plot NDVI was calculated as described previously (Tolley 2023). NDVI was linearly interpolated between flight dates, and plot senescence was quantified as the percent change in NDVI from anthesis to 550 GDD after anthesis. The NDVI curves for all plots in the experiment are shown in Supplemental Figure S1. Yield components from five representative plants of each plot were quantified using an ear photometry platform developed by Corteva Agriscience (Hausmann et al. 2009), formerly Pioneer Hi-bred International, described by Tolley et al. (2021). Yield and other traits from the testcross experiment were estimated as BLUPs within a single-stage mixed-linear model, where genetic effects were partitioned as random effects. As defined previously (Griffing 1956; Lu et al. 2020; Riedelsheimer et al. 2012), the random effect terms for male and female are equal to the general combining ability (GCA) of the inbred lines within this population and the male/female interaction term is equal to the specific combining ability (SCA) of the hybrids. The year/male, year/female, and year/male/female terms were included to account for genotype-by-year variation, ensuring that the GCA and SCA estimates reflect stable performance across the study duration.

$$\begin{aligned} & {\mathrm{Trait}}\sim {\mathrm{mean}}\;\; + ({\mathrm{1|Year}}) + ({\mathrm{1|Year:Rep}}) + ({\mathrm{1|Year:Row}}) + ({\mathrm{1|Year:Range}}) \\ & \quad + ({\mathrm{1|Male}}) + ({\mathrm{1|Female)}} + ({\mathrm{1|Male:Female}}) + (1{\mathrm{|Year:Male)}} \\ & \quad + ({\mathrm{1|Year:Female)}} + ({\mathrm{1|Year:Male:Female)}} \\ \end{aligned}$$

$${\mathrm{TraitBLUP}} = {\mathrm{GrandMean}} + {\mathrm{GCA}}_{{{\mathrm{Male}}}} + {\mathrm{GCA}}_{{{\mathrm{Female}}}} + {\mathrm{SCA}}_{{\mathrm{Male:Female}}}$$

### Genotypic data analysis—imputation and population structure

Genotypic data for the exPVP inbred lines were obtained from a previous study (Beckett et al. 2017). We started with what Beckett describes as the “merged genotype file” containing 349 accessions and 1,281,671 SNPs from multiple sequencing experiments. Using TASSEL 5.0 (Bradbury et al. 2007), SNPs with heterozygous genotypes were set to missing and SNPs with a missing rate of > 20% were removed. Many SNPs were not consistent across datasets, so this filter returned only 351,379 SNPs. From this filtered set, missing data were imputed using the LD KNNi method with 30 high LD sites, 10 nearest neighbors, and 10,000,000 bp as the maximum distance between sites to find LD. Any data that remained missing were imputed using the numerical mean of the five nearest neighbors according to Euclidean distance implemented in TASSEL 5.0 (Bradbury et al. 2007).

Each variety was classified into a heterotic group using admixture analysis (White et al. 2020). The number of ancestral families was set to three because plant breeders widely consider elite material to belong to one of three major families (Stiff Stalk, Non-Stiff Stalk, and Iodent), each containing many sub-families (Beckett et al. 2017; White et al. 2020). The software PLINK 1.9 (Chang et al. 2015) was first used to simultaneously convert the genotypic data into a binary format and filter genotypes to retain SNPs with minor allele frequency of 5% or more. The ADMIXTURE software (Alexander et al. 2009) was used to estimate the proportion of each genotype inherited from each ancestral population. Each variety was classified into the ancestral group, which represented the largest fraction of its genome.

A phylogenetic tree for the exPVP inbred lines was constructed using the method described by Beckett et al. (2017). Briefly, a Nei’s distance matrix between genotypes was calculated using the R package “NAM” (Xavier et al. 2015), and the built-in R function “hclust” was used to perform hierarchical clustering with Ward’s error sum of squares method.

For the genome-wide association, the original SNP genotypes for the 349 maize inbred lines from Beckett et al. (2017) were converted to VCF format using TASSEL v5.0 (Bradbury et al. 2007). We successfully lifted 1,215,360 SNP positions to the B73v4 assembly using CrossMap v0.6.3 (Zhao et al. 2014). We obtained the chain file from MaizeGDB to convert SNP positions from B73v2 to B73v4 (Portwood et al. 2019). After lifting SNPs to B73v4 positions, the SNPs were sorted, ambiguous positions were removed, heterozygous genotypes were set to missing, and only biallelic SNPs were retained. These operations were performed using a suite of functions in *bcftools* (Danecek et al. 2021). The HapMap3 population of 1210 maize accessions and 55,242,281 SNPs was used as the reference sample for imputing SNPs (Bukowski et al. 2018). The normalized SNP data were used for SNP imputation using *beagle v5.1* (Browning and Browning 2016). The window size for imputation was set to 30 Mbp with an overlap of 3.6 Mbp with the previous imputed block. The burn-in iterations of 10 and sampling iterations of 15 were used with the effective population size of 1000 individuals.

### Genome-wide association analysis and putative candidate gene identification

The imputed SNP set was filtered using *vcftools* (Danecek et al. 2011) to retain 20,090,339 SNP positions that had MAF > = 0.05 with no missing genotypes. These SNP genotypes were used for genome-wide association study (GWAS) using R packages *bigstatr* and *bigsnpr*, which account for population structure using principal components (Price et al. 2006; Privé et al. 2018). To identify unique loci associated with each trait, the genome-wide associations across all SNPs in the genome were filtered to retain only the lowest *p* value SNP–trait association in a 250 kb window around each SNP. This was followed by annotating each SNP to the nearest protein-coding genes and miRNA within 125 kb on either side of the SNP. The annotated SNP–gene genome-wide association results for each trait, which passed the conservative Bonferroni-corrected *P* value threshold at *α* = 0.05 (−log_10_ *P* value = 8.6) were used to identify putative candidate genes.

### Single-locus testing of candidate source-sink-regulated senescence genes

The *D8-Mpl* and *D9-1* semidominant mutant alleles in maize have been described previously (Best and Dilkes 2022). We maintained both alleles as heterozygotes in B73 inbred background by repeated outcrossing. Three inbred lines LH60, B95, and PHR58 were crossed as ear-parents to both *D8-Mpl/* + :B73 and *D9-1/* + :B73 pollen to generate F1 hybrids that segregated ~ 1:1 for wild-type and mutant isogenic siblings. The recessive mutant allele of *camouflage1* (*cf1*) was obtained from Dr. David Braun (University of Missouri, Missouri, USA) in B73 genetic background. All genotypes were evaluated during the summer of 2024 in West Lafayette, IN, as triplicate using RCBD paired plot design. One of the paired plots was allowed to open pollinate, and the other received a non-pollination treatment through the methods described above. In each plot, we measured chlorophyll content index from seven representative plants at 425 GDD and 600 GDD using the methods described above.

### Correlation of source-sink-regulated senescence in inbreds with yield and other traits in F1 hybrids

All senescence BLUPs from the inbred female parents were correlated with the yield-related BLUP from the F1 hybrids and tested for statistically significant correlations using ANOVA. Phenotypic correlations were calculated using a model that included fixed effects for the testcross male (Male), heterotic group of the female parent (HeteroticGroup_Female_), the interaction effect between the male tester and the female heterotic group (Male/HeteroticGroup_Female_), and the effect of the female inbred’s senescence, nested within each unique male-by-heterotic-group combination (Male/HeteroticGroup_Female_/SenescenceBLUP). This model structure tests the correlation between inbred senescence and hybrid yield while accounting for the specific combination of the tester and the female genetic background.

$$\begin{aligned} & {\mathrm{Hybrid}}\,{\mathrm{BLUP}} \sim {\mathrm{Male}} + {\mathrm{HeteroticGroup}}_{{{\mathrm{Female}}}} \\ & \; + {\mathrm{Male}}:{\mathrm{HeteroticGroup}}_{{{\mathrm{Female}}}} \, \\ & \; + {\mathrm{Male}}:{\mathrm{HeteroticGroup}}_{{{\mathrm{Female}}}} :{\mathrm{SenescenceBLUP}} \\ \end{aligned}$$

### Statistical analyses

We normalized the phenotypic dataset to remove outliers that were greater than three standard deviations away from the population mean across each experiment. Differences among senescence phenotypes across heterotic families were explored with Tukey’s test using the TukeyHSD implemented in the R stats package using a *p* value threshold of 0.05 (R Core Team 2024). The significance of terms in the mixed-linear models was evaluated using the R package lmerTest (Kuznetsova et al. 2017). Fixed effects were tested using Satterthwaite’s method to calculate denominator degrees-of-freedom and the F-statistic implemented in the *anova* function. Random effects were tested using REML-likelihood ratio tests of model reductions that are implemented in the *ranova* function. Phenotypic Pearson’s correlations of senescence traits in inbred lines and yield-related traits of F1 hybrids were deemed significant using a p value < 0.05.

## Results

### Comparison of senescence traits in the exPVP inbred population

**Fig. 1:**
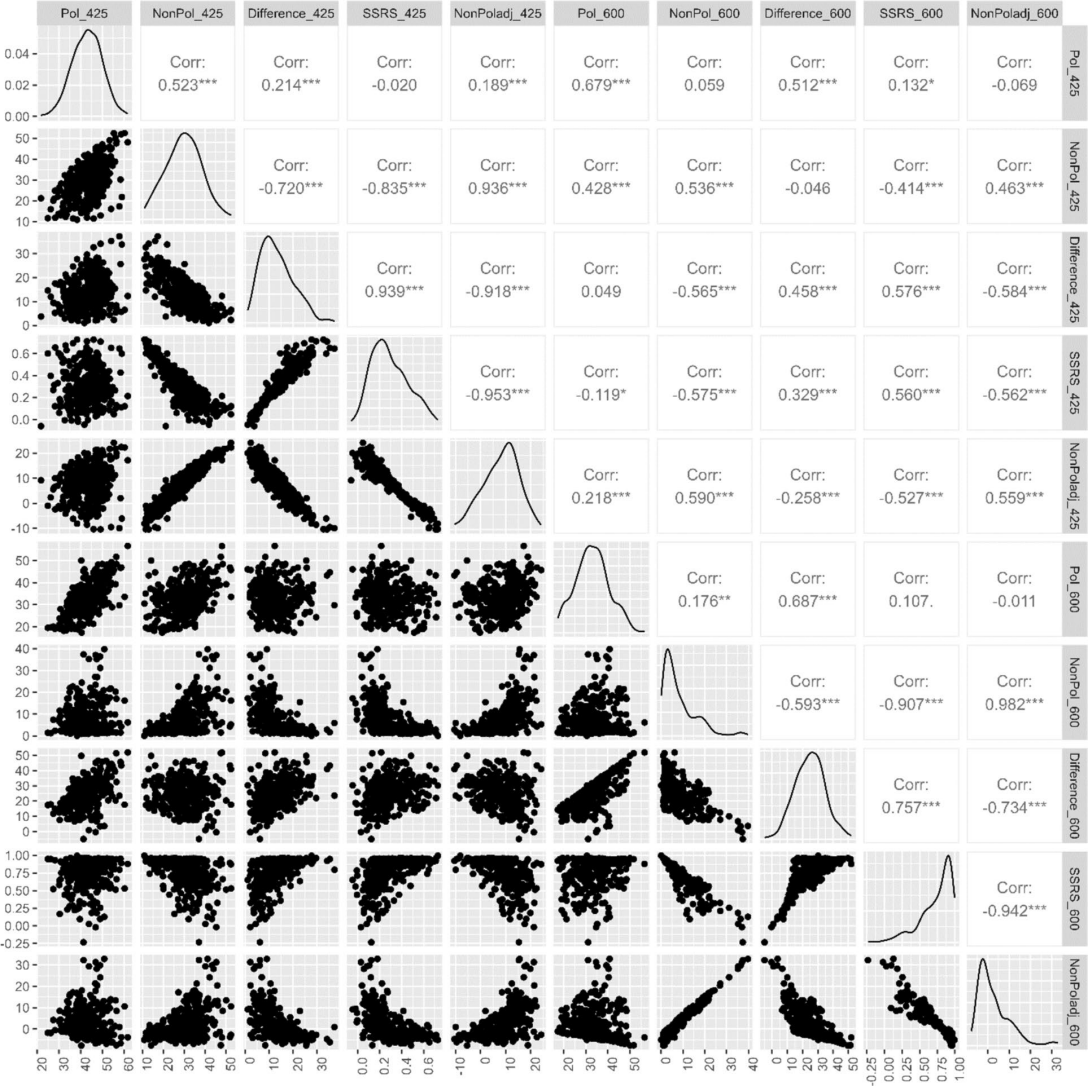
Covariance matrix of senescence traits measured 425 and 600 GDD. Significance of correlations is shown within each panel: “*” 0.05, “**” 0.01, “***” 0.001

To assess the relationships between all senescence phenotypes measured in the exPVP inbred population, we first calculated the Pearson correlation coefficient for all pairwise comparisons. At the earlier timepoint (425 GDD post-anthesis), chlorophyll content of the pollinated (Pol_425) plants was positively correlated (*r* = 0.52) with the chlorophyll content in non-pollinated (NonPol_425) plants (Fig. 1). At the later timepoint (600 GDD post-anthesis), only weak correlation (*r* = 0.18) was observed between Pol_600 and NonPol_600. Across the two measurement timepoints, Pol_425 showed a high positive correlation with Pol_600 and NonPol_425 showed a high positive correlation with NonPol_600. However, the chlorophyll content in the pollinated plants at the earlier timepoint (425 GDD) showed only a weak positive correlation with the non-pollinated plants at 600 GDD. This suggests a strong senescence response to non-pollination, which is independent from the chlorophyll content of pollinated plants at 425 GDD and results in severe reduction in chlorophyll content by 600 GDD. The senescence traits derived from direct phenotypic measurements showed strong correlation with each other and a weak-to-no correlation with chlorophyll content in pollinated plants. This is expected because these calculated traits are designed to remove the variations in baseline chlorophyll across genotypes. As expected for derived traits, strong correlation was observed among the traits quantifying senescence response at a single timepoint (NonPol with NonPoladj, SSRS, and Difference). For instance, at 425 GDD, the correlation coefficients of NonPol with NonPoladj, SSRS, and Difference are 0.939, −0.951, and −0.918. Traits were still highly correlated at 600 GDD, but the complete senescence of some genotypes by this timepoint thresholded trait values. We then calculated the broad-sense heritability (H^2^) of all senescence traits in the exPVP inbred population. The H^2^ estimates ranged from 0.56 to 0.82 across all pollinated and non-pollinated traits measured at a single timepoint (Table 1). The high heritability estimates especially across senescence traits suggest a strong genetic component.

Traits based on the rate of senescence had lower heritability than the traits calculated from measurements at a single timepoint and also had low correlation with traits from a single timepoint (Figure S2 and S3). Within the rate-related traits, all traits were highly correlated except the rate of senescence for pollinated plants, which had a very low heritability and was not correlated with any other traits.

The threshold effect at 600 GDD due to complete loss of chlorophyll limits the comparison of many genotypes. Therefore, to draw biological insights on the relationship between source-sink senescence and yield traits in testcross hybrids, we focused on the earlier timepoint of 425 GDD, which displayed continuous variation in senescence across genotypes. We specifically used NonPoladj trait because it was highly correlated with the other senescence traits and had high broad-sense heritability.

### Distribution of senescence phenotypes across heterotic groups

**Fig. 2:**
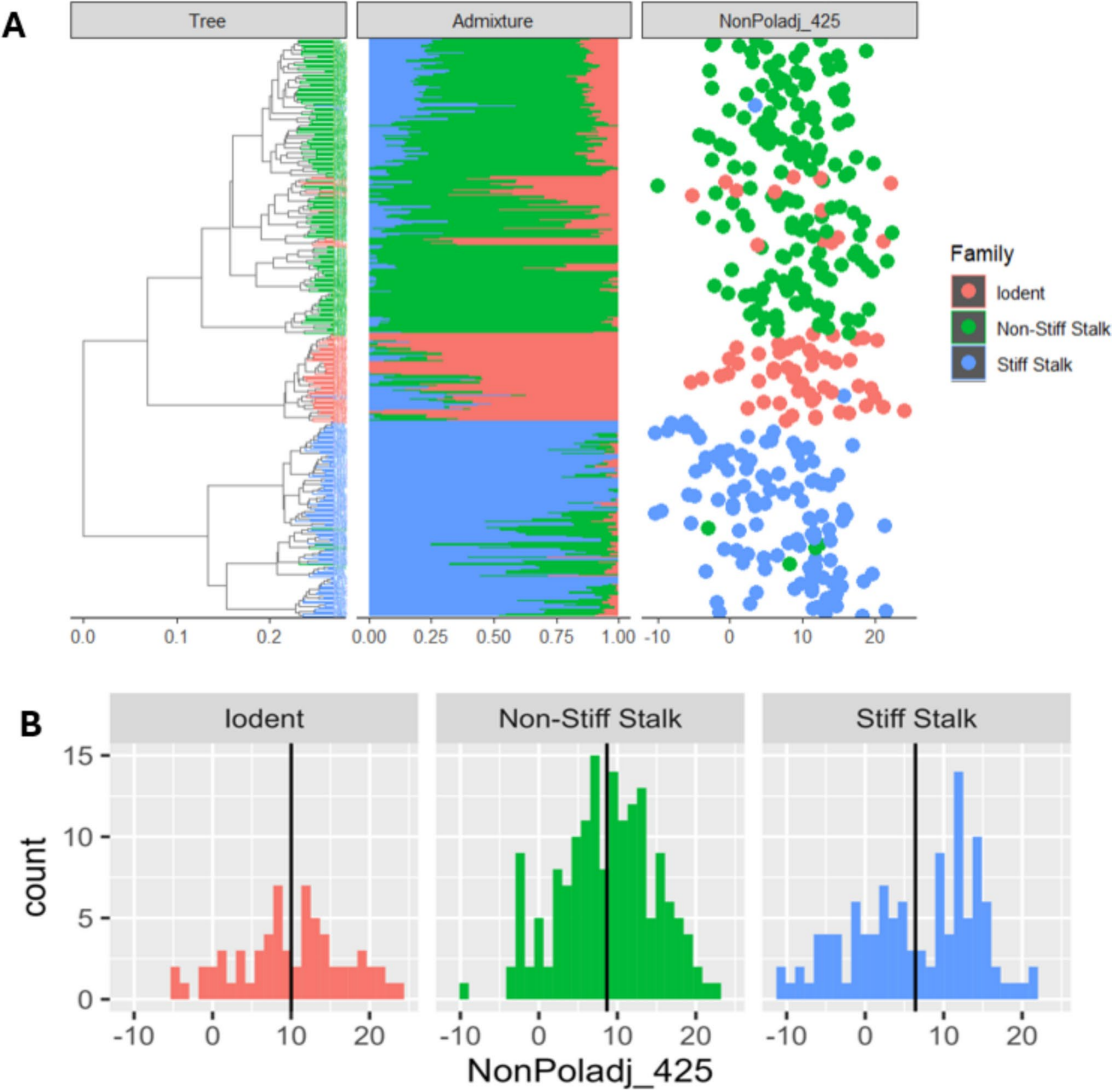
Phylogenetic distribution of senescence phenotypes. **A** Dendrogram of exPVP lines paired with admixture plot and plot of the senescence response to sink disruption (NonPoladj_425). The genotypes are spatially plotted according to the dendrogram and colored based on the admixture family representing the largest fraction of the genome. Smaller values of NonPoladj indicate stronger senescence response. **B** Histogram of NonPoladj BLUP at 425 DD (NonPoladj_425) across heterotic groups. Vertical lines represent the mean of each heterotic group

To understand the distribution of senescence phenotypes in the exPVP population, we studied the variation in senescence in response to sink disruption across the genetic structure of the population using NonPoladj BLUP at 425 GDD (Fig. 2). Lower NonPoladj BLUP values represent lower chlorophyll content and, hence, indicate a stronger senescence response to sink disruption. We observe that the senescence trait is widely distributed across heterotic groups with no branch of the dendrogram clearly exhibiting an extreme senescence phenotype. The genotypes are spatially arranged according to the dendrogram but colored based on the admixture family representing the largest fraction of the genome. In general, the phylogenetic tree and admixture align, but there are some exceptions. The dendrogram is based on identity by state, while in reality, the inbred lines contain a diverse admixture of several ancestral haplotypes. The “mis-colored” varieties are more similar by state to the dendrogram branch they are plotted with but contain a diverse admix of multiple ancestors with a slightly larger proportion belonging to a family from another part of the dendrogram. This is expected in an elite germplasm pool where families are regularly mixed to produce better germplasm. There are several detailed analyses of the evolution of commercial maize germplasm that may inform the interested reader (Beckett et al. 2017; Mikel and Dudley 2006; Troyer 1999; White et al. 2020).

We further compared the distribution of source-sink-regulated senescence within heterotic groups. The mean BLUP for NonPoladj at 425GDD and standard deviation by heterotic group were as follows: Iodent (*μ* = 10.0, *σ* = 6.9), Non-Stiff Stalk (*μ* = 8.7, *σ* = 6.1), and Stiff Stalk (*μ* = 6.4, *σ* = 7.7). The Stiff Stalk group displayed wider variation in NonPoladj_425 and carried inbred lines with strongest senescence response. This is reflected in the lower mean of Stiff Stalk inbred lines compared to the non-Stiff Stalk and Iodent populations (two-tailed Tukey’s test with *P* < = 0.05). No significant difference in population means was observed between the Iodent and Non-Stiff Stalk populations.

### Correlation of source-sink senescence with yield

**Fig. 3:**
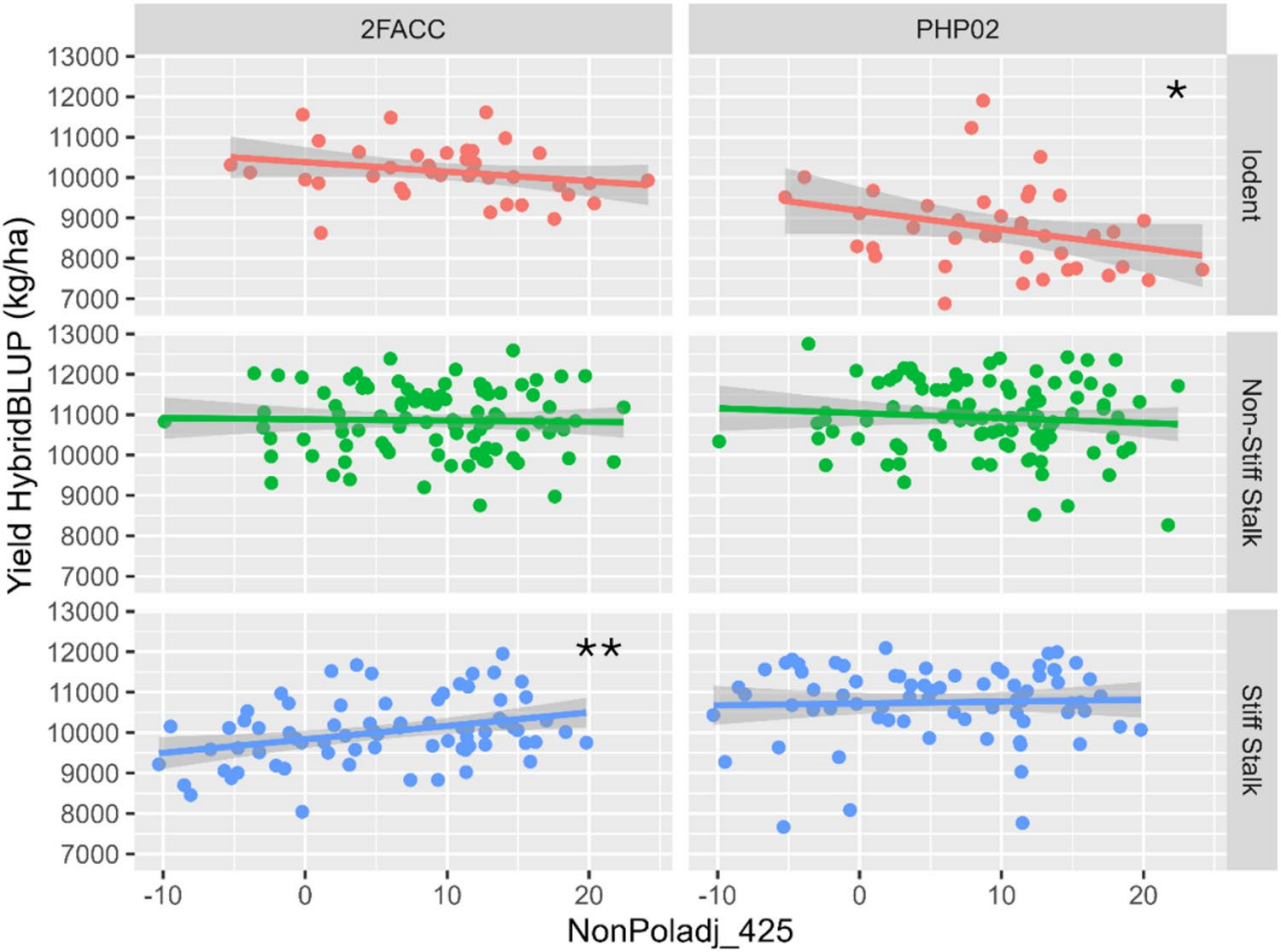
Correlation of source-sink senescence with yield. The BLUP for NonPoladj_425 is plotted with the BLUP for hybrid yield (kg/ha) split by heterotic group and across the Stiff Stalk male tester (2FACC) and an Iodent tester (PHP02). Smaller values of NonPoladj indicate a stronger senescence response to sink disruption. The trend is only significant when the male tester is from the same heterotic family as the female inbred lines. Significance of correlations is shown within each panel: ‘*’ 0.05, ‘**’ 0.01, ‘***’ 0.001

Statistically significant correlations were found between the yield of hybrid testcrosses and the source-sink senescence traits of their inbred parents. Figure 3 shows a plot of inbred source-sink senescence (NonPoladj_425) with the hybrid yield BLUP of the female parents in crosses to a Stiff Stalk male tester (2FACC) and an Iodent tester (PHP02). Significant correlations were observed in the crosses of Iodent inbreds to the Iodent tester PHP02 (*p* = 0.023) and crosses of Stiff Stalk inbreds to the Stiff Stalk tester 2FACC (*p* = 0.009). This is notable because the trend is only significant when the male tester is from the same heterotic family as the female inbred lines. Commercial breeding programs typically only make crosses between two different families because heterosis is not observed in crosses between closely related individuals. The intra-heterotic pool crosses within the Iodent group displayed a yield increase of 1100 kg/ha from the most senescent to least senescent inbred x PHP02 crosses, while the intra-heterotic pool crosses within the Stiff Stalk group show a yield decrease of 800 kg/ha from the most senescent to least senescent inbred x 2FACC crosses. Crosses made between parents from different heterotic groups did not show any significant phenotypic correlation between NonPoladj_425 and yield. The other senescence traits measured at 425 GDD are highly correlated with NonPoladj and show similar trends to Fig. 3. The senescence traits measured at 600 GDD also mirror the trends observed at 425 GDD. Rate of senescence traits was not significantly correlated with yield. Correlation results and plots for all traits can be found in the supplementary materials “TCZm Trait Models.”

### Correlation of source-sink senescence with yield components

#### Iodent heterotic group

**Fig. 4:**
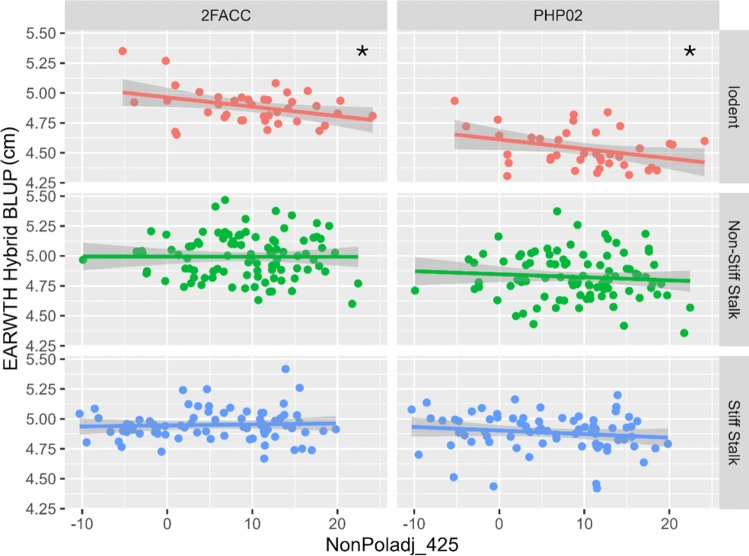
Correlation of source-sink senescence and ear width. The BLUP for the NonPoladj trait is plotted with the BLUP for EARWTH (cm) split by heterotic group and across the Stiff Stalk male tester (2FACC) and an Iodent tester (PHP02). Smaller values of NonPoladj indicate a stronger senescence response. The trend is statistically significant for the Iodent population with both testers but is not significant for the Stiff Stalks nor Non-Stiff Stalks. Significance of correlations is shown within each panel: ‘*’ 0.05, ‘**’ 0.01, ‘***’ 0.001

Within the Iodents, there were significant trends in ear morphology which could explain the observed trends in yield. Between crosses of inbred lines from the Iodent heterotic pool to the Iodent inbred tester PHP02, all source-sink senescence traits were significantly correlated with increases in ear width (EARWTH) which translated into significant increases in ear area (EARAREA) and ear volume (EARVOL). The correlations with EARWTH are shown in Fig. 4. There was not a significant relationship with ear length, number of kernel rows, or total kernels per ear, and there was an inverse relationship between source-sink senescence and kernel width. Stronger senescence response was correlated with the higher levels of scatter grain, which may be caused by larger ear sizes. Stronger source-sink senescence response in inbred parents was correlated with delays in senescence within the hybrid crosses.

The hybrid crosses between Iodent inbred lines and the stiff stalk inbred 2FACC showed a positive correlation between senescence and kernels per row that also translated into a positive correlation with total kernels per ear. All source-sink senescence traits were significantly correlated with increases in EARWTH and ear central width (EARCW), which translated into significant increases in EARVOL. The crosses to 2FACC showed an inverse relationship between source-sink senescence and kernel width along with no increase in ear length, which could explain why the increases in kernel numbers did not lead to increases in yield.

#### Stiff stalk heterotic group

**Fig. 5:**
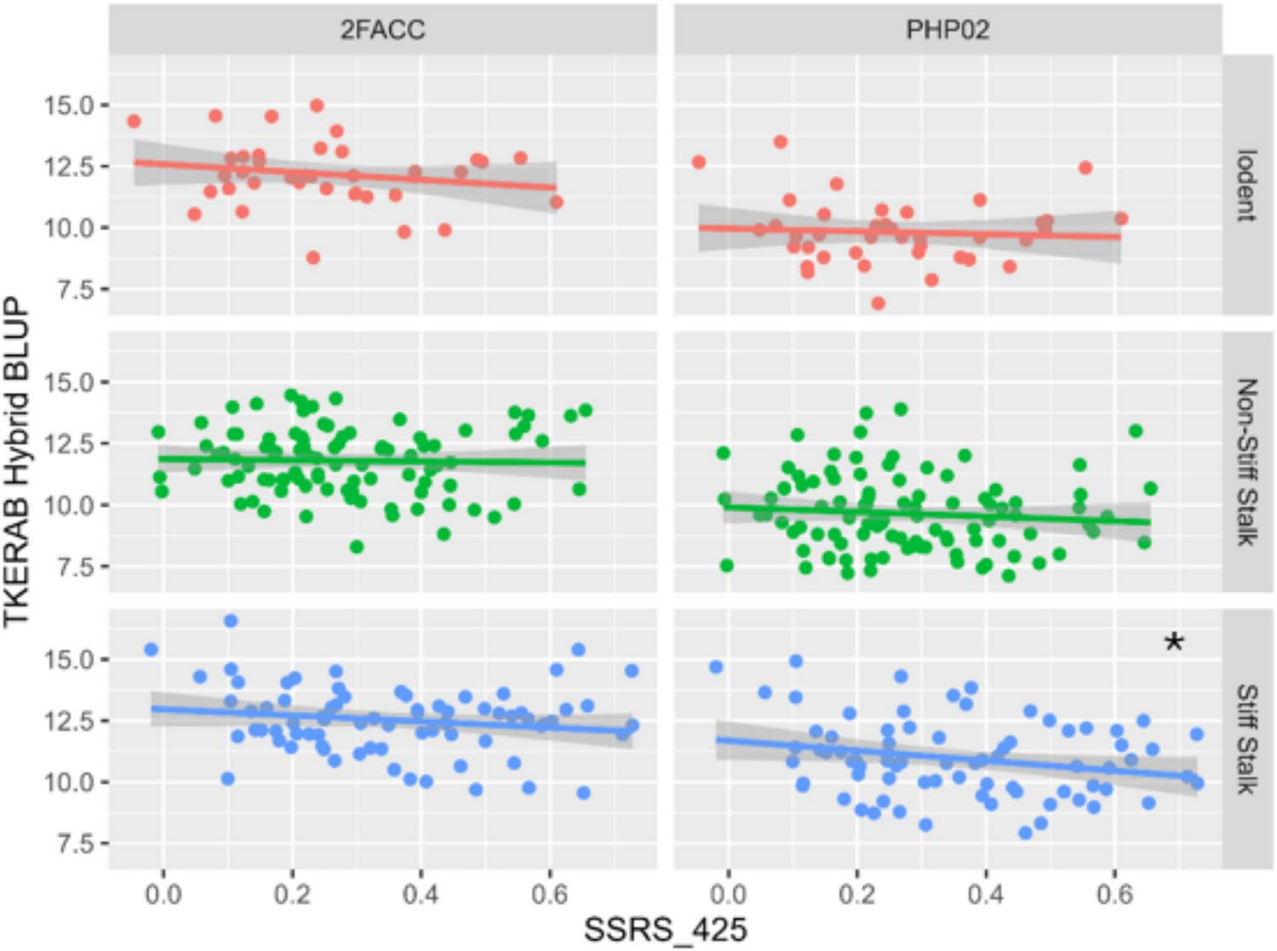
Correlation of senescence and kernel abortion. The BLUP for SSRS_425 is plotted with the BLUP for kernel abortion (TKERAB) split by heterotic group and across the Stiff Stalk male tester (2FACC) and an Iodent tester (PHP02). Larger values of SSRS indicate a stronger senescence response. The trend is consistent across all heterotic combinations but only statistically significant for the crosses between Stiff Stalks and PHP02. Significance of correlations is shown within each panel: ‘*’ 0.05, ‘**’ 0.01, ‘***’ 0.001

Significant correlations between source-sink senescence traits and decreases in kernel abortion (TKERAB) were observed in F1 testcrosses of Stiff Stalks to PHP02 across a variety of traits and timepoints. Figure 5 shows the correlations between SSRS measured at 425 GDD and the TKERAB hybrid BLUP. The direction of the trend is consistent across all other hybrid combinations at a nonsignificant level. We would expect less abortion to translate into either more kernels per ear or smaller ears, which would offset the impact on kernel abortion on total kernel number, but neither is observed at a statistically significant level. There are no other trends within the Stiff Stalk family that are consistent across multiple senescence traits.

#### Non-Stiff Stalk heterotic group

In crosses to 2FACC, source-sink senescence response in the testcrosses with the Non-Stiff Stalk heterotic pool was correlated with larger kernel length, which translates to significant increases in kernel perimeter. However, source-sink senescence response and faster rates of senescence response in Non-Stiff Stalks are also correlated with fewer kernel rows in the hybrid testcrosses. As there is no significant correlation between source-sink senescence response and yield within the Non-Stiff Stalk population, it seems that the opposing responses of increased kernel size and decreased number of kernel rows counterbalance each other. In crosses to PHP02, the higher chlorophyll levels in pollinated plants and faster rates of senescence are correlated with increased kernel fill and less scatter grain. However, these relationships are not associated with significant increases in yield. Across both testcross parents, higher chlorophyll levels in pollinated plants from the Non-Stiff Stalk population are correlated with less senescence in F1 hybrid populations. With crosses to PHP02, this trend is more pronounced and also observed with chlorophyll levels in non-pollinated plants.

### Genome-wide association study of senescence traits in exPVP inbreds

**Fig. 6:**
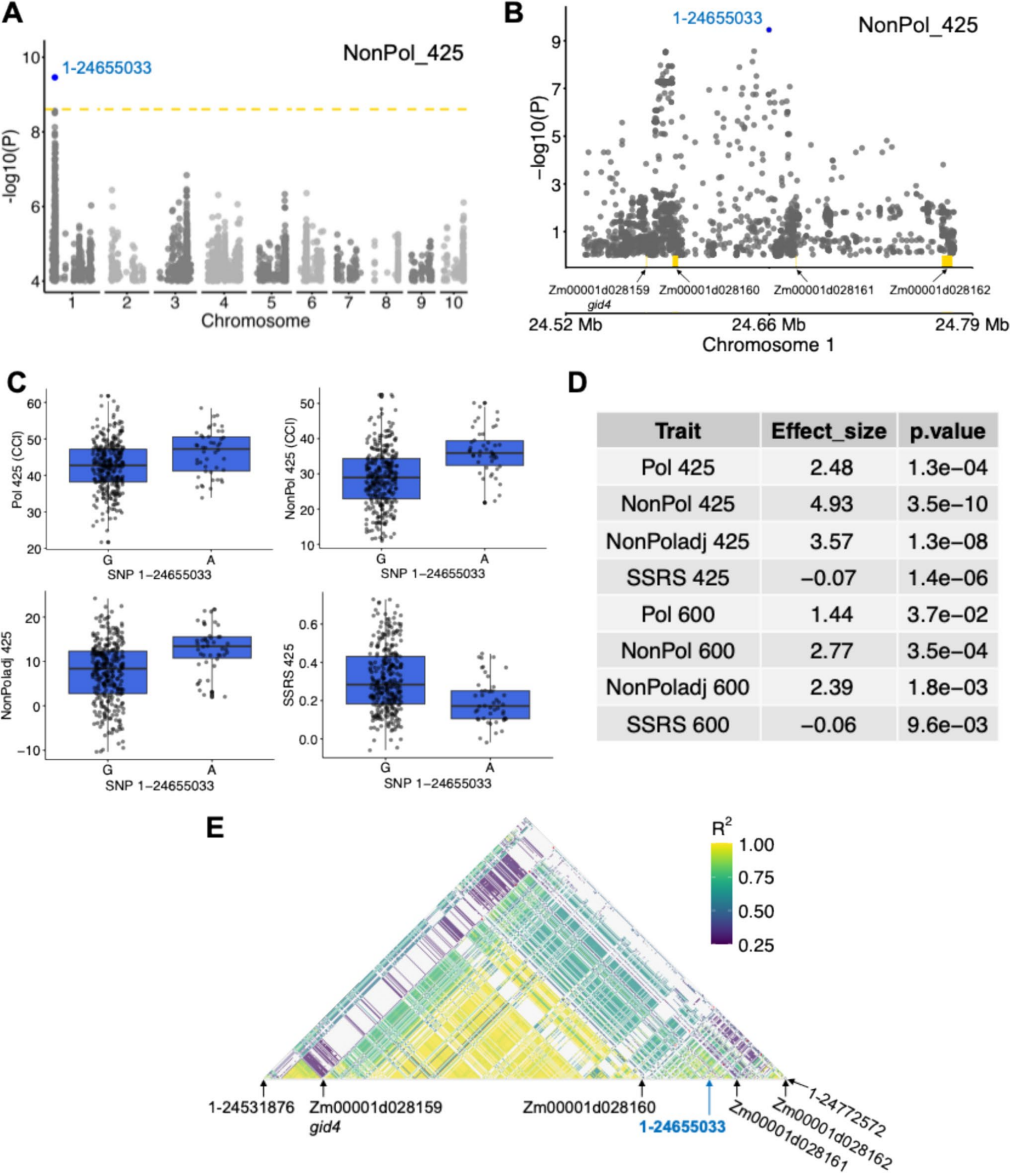
Genome-wide association identified *gid4* as a putative candidate for source-sink-regulated senescence in exPVP inbred lines. **A** Genome-wide association of chlorophyll content index at 425 DAA in non-pollinated plants in the exPVP inbred panel. Dashed golden line is the Bonferroni-corrected genome-wide threshold (−log_10_ *p* value = 8.6) and the significant association with SNP 1–24,655,033 is highlighted. **B** Zoomed in view of the 250 kb genomic region flanking the SNP 1–24,655,033 association, with all genes in the interval shown in yellow. **C** Trait distribution at 425 DAA in the individuals carrying the reference “G” and non-reference “A” alleles at SNP 1–24,655,033. **D **Allele effect and p value across all traits at SNP 1–24,655,033. **E** Linkage disequilibrium (*R*^*2*^) at the locus represented in panel B using all the SNPs with *p* value < 0.01. White regions in the pairwise comparisons in the heat plot had *R*^2^ < 0.25

To understand the genetic architecture of source-sink-regulated senescence in the exPVP population, we carried out a genome-wide association study (GWAS) of all senescence traits. At the Bonferroni threshold, GWAS identified five unlinked loci with SNP–trait associations (Fig. 6). The top SNP from these loci is located on chromosome 1 at 24,655,033 bp (SNP 1–24,655,033), chromosome 3 at 115,601,313 bp (SNP 3–115,601,313) and 157,623,549 bp (SNP 3–157,623,549), chromosome 4 at 241,458,461 bp (SNP 4–241,458,461), and chromosome 6 at 142,491,809 bp (SNP 6–142,491,809). Given the high phenotypic correlation between senescence traits, each locus was associated with multiple traits (Table S2). Protein-coding genes and miRNA within 125 kb on either side of these SNP are shown in Table S3.

The SNP 1–24,655,033 was associated with senescence induced by non-pollination at 425 GDD post-anthesis. One direct (NonPol_425) and three derived trait measurements (NonPoladj_425, Difference_425, and SSRS_425) at 425 GDD were associated with this SNP, with NonPol_425 showing the strongest association (Fig. 6A, D). The 125 kb genomic region flanking SNP 1–24,655,033 contains four protein-coding genes (Fig. 6B). The non-reference allele at this QTL is associated with higher chlorophyll content in non-pollinated plants, and a weaker allele effect in the same direction was also observed in pollinated plants at both timepoints (Fig. 6C, D). To determine the linkage among SNPs at this QTL, we calculated pairwise linkage disequilibrium (Fig. 6E). The *R*^2^ values between SNP 1–24,655,033 and the SNP tagging each gene were *gibberellin-insensitive dwarf protein homolog4* (*gid4;* Zm00001d028159; SNP 1–24,572,076; R^2^ = 0.35), *semi-rolled leaf5* (*srl5;* Zm00001d028160; SNP 1–24,589,942; *R*^2^ = 0.04), Zm00001d028161 (SNP 1–24,673,331; R^2^ = 0.30), and *sulfate permease1* (*sfp1;* Zm00001d028162; SNP 1–24,772,545; *R*^*2*^ = 0.53). These are putatitive candidate genes, which require functional validation.

**Fig. 7:**
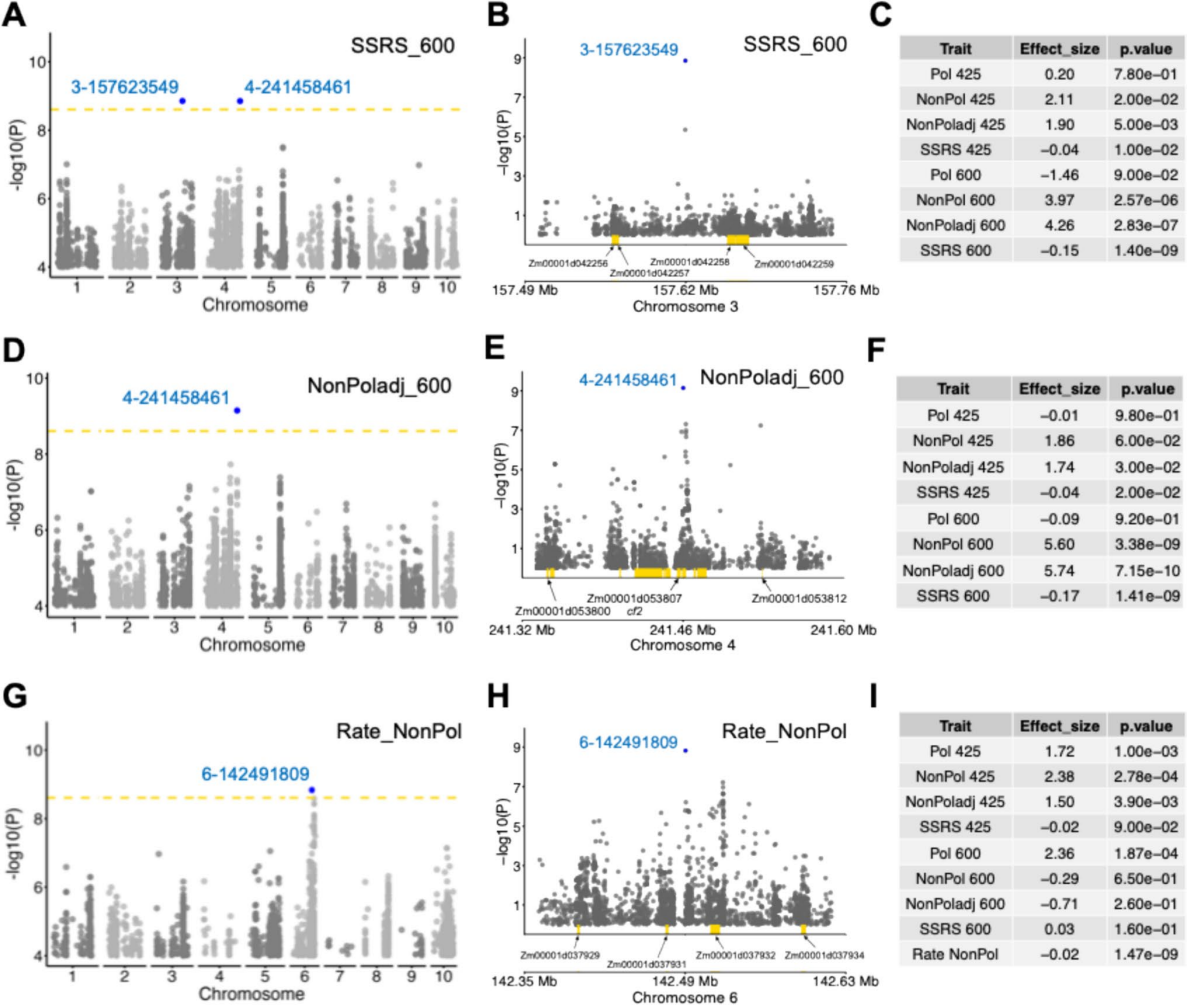
Genome-wide association identified multiple loci associated with source-sink-regulated senescence in exPVP inbred lines at Bonferroni threshold (yellow line; −log_10_ *p* value = 8.6). **A** Genome-wide association of SSRS_600, and **B** the 125 kb genomic region flanking the top SNP 3–157,623,549 along with nearby genes (yellow box) in this interval. **C** Allele effect and *p* value across select traits at SNP 3–157,623,549. **D** Genome-wide association of adjusted chlorophyll index at 600 GDD in non-pollinated plants, and **E** Zoomed in view of the 125 kb genomic region flanking the top SNP 4–241,458,461 along with nearby genes. **F** Allele effect and *p* value across select traits at SNP 4–241,458,461. **G** Genome-wide association of Rate_NonPol, and **H** the 125 kb genomic region flanking the top SNP 6–142,491,809. **I** Allele effect and p value across select traits at SNP 6–142,491,809

The SNP 3–115,601,313 was associated with the pollinated trait values at both 425 and 600 GDD post-anthesis and the difference trait at 600 GDD (Table S2). The 125 kb genomic region flanking the SNP contained three protein-coding genes, with only two expressed genes. The SNP 3–157,623,549 was associated with multiple senescence traits at 600 GDD post-anthesis (Fig. 7A–C and Table S2) with SSRS_600 exhibiting the most significant association. The fourth association with SNP 4–241,458,461 was specific to senescence traits at 600 GDD post-anthesis (Fig. 7D–F). One direct (NonPol_600) and three indirect trait measurements (NonPoladj_600, Difference_600, and SSRS_600) at 600 GDD were associated with the SNP 4–241,458,461 (Table S2). The fifth SNP association at 6–142,491,809 was unique as it was not associated with any SSRS phenotypes measured at a single timepoint but was associated with rate of senescence measured by different traits (Rate_NonPol, Rate_NonPoladj, Rate_Difference, and Rate_SSRS) across 425 and 600 GDD post-anthesis (Fig. 7G–I and Table S2).

### Single-locus test of candidate genes associated with senescence

**Fig. 8:**
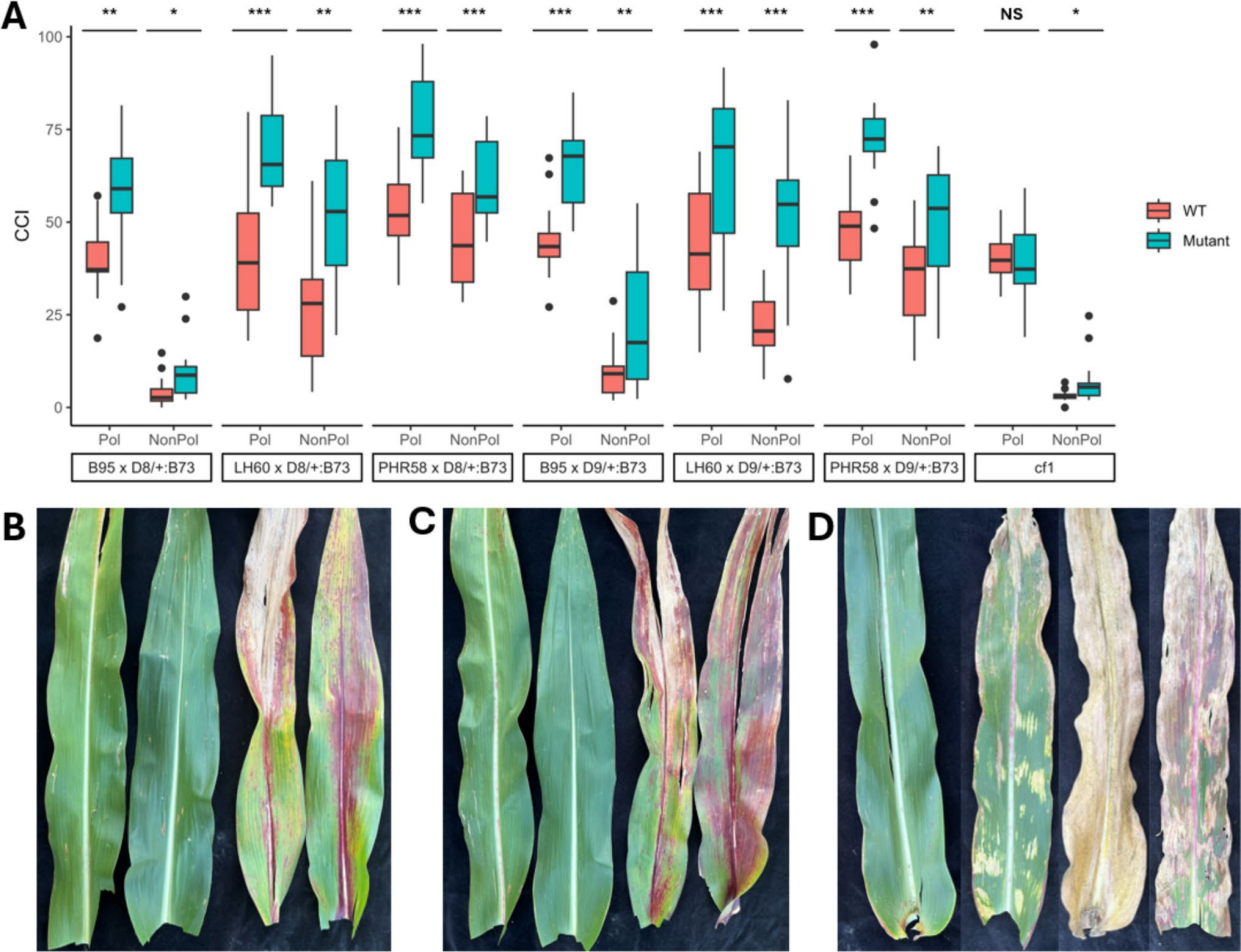
Single-locus testing of source-sink senescence candidate genes. (**A**) Boxplot of chlorophyll content index (CCI) for wild type (WT) and mutants measured at 425 GDD after anthesis under both pollinated and non-pollinated treatments. Significance of *t* test for difference in means between each WT and mutant pair are shown as: “*” 0.05, “**” 0.01, “***” 0.001. **B**–**D** One representative leaf sampled at 425 GDD after anthesis (left to right) across pollinated wild-type, pollinated mutant, non-pollinated wild-type, and non-pollinated mutant plants from following pedigrees **B** LH60 x *D8-Mpl*/ + :B73 **C** LH60 x *D9-1*/ + :B73 **D** B73 and *cf1*:B73. All pedigrees with D8 and D9 mutants are shown together in Supplementary Figure S5

In the GWAS analysis of the exPVP inbred lines outlined above, gibberellic acid (GA) and porphyrin biosynthesis were identified as two candidate biochemical pathways that could possibly alter leaf senescence. The SNP 1–24,655,033 is linked to a GA receptor *gid4* and displayed strong association with non-pollination-induced senescence at 425 GDD post-anthesis (Fig. 6). DELLA domain transcription factors encoding GA signaling repressors are known to delay leaf senescence, whereas GA receptor activity hastens leaf senescence potentially due to degradation of DELLA proteins via proteasomal degradation in a GA-dependent manner (Chen et al. 2014). Maize carries two DELLA domain paralogs, *dwarf8* and *dwarf9*, that repress GA signaling and previous work in maize described semidominant alleles at both loci, which enhance DELLA repression of GA signaling (Best and Dilkes 2022). The impact of semidominant *D8-mpl* and *D9-1* mutant alleles was evaluated in senescence permissible exPVP x B73 F1 hybrid backgrounds. In each cross, the exPVP inbred was crossed as an ear-parent to the pollen from the mutant heterozygote in the B73 background. Each F1 family segregated ~ 1:1 for isogenic mutant and wild-type F1 hybrids which provided a perfect isogenic control to test the effect of mutant allele on senescence. Isogenic wild-type and mutant F1 hybrids reached reproductive maturity at the same time. The *D8-mpl/* + and *D9-1/* + F1 hybrids in all three genetic backgrounds exhibited higher chlorophyll levels than the isogenic wild-type hybrids (Fig. 8). The wild-type hybrids in each combination showed a strong senescence phenotype in response to non-pollination at 425 GDD. Mutant F1 hybrid siblings containing either *D8-mpl* or *D9-1* allele also displayed a strong senescence response to non-pollination and lost more chlorophyll in absolute terms than their wild-type siblings. However, the non-pollinated mutant plants at 425 GDD were still greener than their wild-type non-pollinated counterparts likely due to the higher initial levels of chlorophyll in these mutants. At 600 GDD, the mutant F1 hybrid siblings were still significantly greener than wild-type F1 hybrid siblings when fully pollinated, but both wild type and mutants have completely senesced in response to non-pollination (Figure S4). It seems that observed differences in *D8-Mpl* and *D9-1* mutants that carry a dominant-negative alleles are due to an elevation in chlorophyll levels rather than an attenuation of the senescence response to sink disruption. This observation is consistent with previous reports of staygreen trait in maize showing a strong positive correlation with SSRS (Kumar et al. 2019). Moreover, in our GWAS analysis, the alleles associated with higher chlorophyll under non-pollinated conditions were also weakly associated with higher chlorophyll in pollinated conditions at both timepoints.

The effect of porphyrin biosynthesis pathway was also evaluated for its influence on senescence induced by non-pollination. The SNP 4–241,458,461, associated with senescence at 600 GDD post-anthesis, was linked to a paralog of *camouflage1* that encodes chloroplast-localized porphobilinogen deaminase enzyme (Fig. 7D–F). The block in porphyrin biosynthesis is proposed to cause a reduction in heme biosynthesis and therefore reduces ROS scavenging capacity (Huang et al. 2009). Previous literature has hypothesized that ROS scavenging is a critical mechanism driving the senescence response to sink disruption in maize (Kumar et al. 2023). We used a recessive mutant allele of *cf1* (Huang et al. 2009) in a senescence permissible B73 background to test the impact of the porphyrin biosynthesis pathway on senescence response to sink disruption. The *cf1* mutants form non-clonal necrotic sectors early in leaf development. These sectors undergo premature cell death well before we applied the non-pollination treatment, so we specifically measured the non-necrotic sectors of the *cf1* mutants. Even with globally reduced PGBD enzyme activity and disruption of the porphyrin biosynthesis pathway, the senescence in *cf1* mutants in non-necrotic sectors was not enhanced by non-pollination with respect to wild-type B73 siblings (Fig. 8). On the contrary, slightly higher chlorophyll levels were observed in *cf1* mutants after non-pollination at 425 GDD. This demonstrates that disruption of porphyrin biosynthesis in *cf1* does not enhance the senescence response to non-pollination.

### Loci associated with source-sink-regulated senescence in inbreds do not correlate with hybrid yield traits

The markers identified by the GWAS for senescence in inbreds were tested for association with hybrid yield phenotypes using a single factor analysis. The test was nested within male tester and heterotic group and used a *P* value threshold at *α* = 0.05 corrected for the number of independent tests (−log_10_ *P* value = 4.04). Three of the five markers (SNP 1–24,655,033, SNP 3–157,623,549, and SNP 4–241,458,461) were monomorphic within the Iodent population so it was not possible to test the effects of these SNP on hybrid traits within the Iodent population. The strongest correlations identified, PHTKPR (−log_10_ *P* value = 3.89), EARRC (−log_10_ *P* value = 3.56), and Yield (−log_10_ *P* value = 2.57), did not pass the significance threshold.

The SNPs and loci identified for NonPoladj_425 in exPVP inbreds were also evaluated for association with SNP for yield and other traits in the hybrid population. To enable this analysis, GWAS were performed for yield and all other traits in both exPVP x PHP02 and exPVP x 2FACC hybrid populations. An arbitrary threshold of *P* value < 10−4 was used to eliminate spurious associations across all traits and SNP were clustered by loci, where locus was defined as a 250 kb genomic region flanking the lowest *P* value SNP. In PHP02 testcrosses, the number of SNPs associated at *P* value < 10−4 with any given trait ranged from 2817 to 26,535 SNPs that corresponded to 317–1986 unique loci (Table S4). In the 2FACC testcrosses, the range of significant SNPs at *P* value < 10−4 was 2740–27783 that corresponded to 397–1743 unique loci (Table S5). Senescence-associated SNPs (SAS) were identified using the same *P* value threshold. A total of 9671 SAS representing 373 unique loci were associated with the NonPoladj_425 in the exPVP inbred population. In the PHP02 testcross population, the average number of significant SNPs common in any pairwise comparison with NonPoladj_425 ranged from 0 to 45, and this represented a maximum of three loci. Similar analysis in the 2FACC testcross population returned between 0 and 3 common SNPs that represented a maximum of two loci. These results were below the expected overlap by random chance. This demonstrates that additive effects for source-sink-regulated senescence in the inbred panel were not detectable for yield and other traits in the two F1 hybrid populations.

## Discussion

### Distribution of source-sink-regulated senescence phenotypes in exPVP germplasm

We observed a wide distribution of highly heritable senescence responses to sink disruption across heterotic families and sub-families in a population of exPVP varieties representing elite maize germplasm. This confirms the results from previous studies (Crafts-Brandner et al. 1984; Kumar et al. 2019; Willman et al. 1987) and suggests that the trait is descended from a common ancestor that predates heterotic families or is the result of convergent evolution. The broad, continuous distribution of the trait observed along with multiple significant loci identified by GWA implies a polygenic control with many additive effects. This is consistent with models proposed in the literature (Kumar et al. 2023) where premature senescence results from excess sugar accumulation causing a complex cascade of sugar-senescence signaling, accumulation of reactive oxygen species, and attempts to sequester sugar in alternate sinks such as stalks and roots.

Almost all 342 lines in this study exhibited a senescence response to sink disruption, and none of the lines showed an increase in CCI due to sink disruption. Previous studies have discussed source-sink senescence in terms of senescent and non-senescent varieties with Mo17 inbred line referred to as a non-senescent and B73 as a senescent inbred line. However, we observed that Mo17 is more senescent than the average exPVP line and more senescent than the average Non-Stiff Stalk inbred. We identified multiple non-senescent lines would serve as a better control than Mo17 in the exPVP population such as MBST (Non-Stiff Stalk), ICI986 (Iodent), and FR19 (Stiff Stalk). The senescence BLUP for all exPVP lines is included in the Supplementary Materials and can be used for genotype selection in future experiments. Senecence phenotypes are environmentally dependent, and we recommend multi-environment replication for future investigation.

### Relationship of senescence traits with yield, staygreen, and ear photometry traits

The yield impacts of source-sink-regulated senescence are dependent on genetic background. Source-sink senescence traits of inbred parents are not significantly correlated with yield in crosses between two different heterotic families. This explains why the trait has not experienced convergent selection in modern breeding and still has a wide distribution of phenotypes in commercial germplasm evaluated in this work and previously published studies (Kumar et al. 2019, 2023; Willman et al. 1987).

Across all three heterotic families, strength of parental source-sink senescence response was positively correlated with increased kernel numbers in hybrid testcrosses although the result was only statistically significant within the Iodent family. This leads to the hypothesis that a strong source-sink senescence phenotype could increase evolutionary fitness by increasing progeny number. Since the source-sink senescence phenotype is not correlated with yield in inter-family heterotic crosses, the trait was not selected against in commercial breeding programs and remains widely distributed throughout commercial germplasm.

Within the Iodent-by-Iodent F1 hybrids, source-sink-regulated senescence is positively correlated with larger ear size in hybrids and increased total number of kernels per ear and total number of rows. As would be expected, yields are also positively correlated with source-sink senescence in the F1 hybrid populations with the Iodent testers. Within Stiff Stalk-by-Stiff Stalk F1 hybrids, yield is negatively correlated with strength of the source-sink senescence response. The decrease in yields observed within the Stiff Stalk family could not be explained by the yield components from the ear photometry system used in this study. Within the Stiff Stalk-by-Stiff Stalk hybrids, ear size remained the same and kernel abortion was significantly decreased with stronger senescence phenotypes. Total kernels per ear were also higher, but at a nonsignificant level. This would require a negative correlation between source-sink senescence and kernel weight. We expected source-sink senescence to affect kernel fill and kernel mass because of the sugar signaling and photosynthesis regulation demonstrated by previous studies (Kumar et al. 2019; Sekhon et al. 2012). However, estimates of kernel mass from the ear photometry system did not show significant trends. The system we used does not assess kernel mass directly, and the correlation of manually measured kernel weights and ear photometry estimations of kernel weights was low accuracy (*r* = 0.51) when the system was originally calibrated (Tolley et al. 2021). This low accuracy is understandable because the system is based on photographing whole ears, which makes it impossible to directly detect the depth of kernel fill. Future work with direct measurement of kernel mass may show how yields are reduced within the Stiff Stalk-by-Stiff Stalk hybrids.

We expected parental source-sink-regulated senescence to be correlated with hybrid senescence at the end of the grain filling period and staygreen. Our ability to quantify the senescence of hybrids was limited to 550 GDD after anthesis due to dates of UAV flights. Significant changes in NDVI were observed during these times (Figure S1) but the relationships with post-flowering senescence in hybrids and parental source-sink senescence were complex. Within the Non-Stiff Stalks, higher levels of chlorophyll in pollinated and non-pollinated parents were correlated with less senescence in the hybrids 550 GDD post-flowering. This implies that the two forms of senescence are linked within the Non-Stiff Stalk background. Within the Iodents, the senescence types were inversely correlated. Inbred parents that senesce more severely in response to sink disruption produce hybrids, which are less likely to senesce 550 GDD post-flowering. It is unclear why this relationship exists and may be caused by another senescence mechanism acting within the Iodent family. In their study of the relationship between ear-removal-induced leaf senescence and green score in maize, Willman et al. also found that source-sink senescence and staygreen were positively and negatively correlated depending on genetic background (Willman et al. 1987). Kumar et al. found relationships between both source-sink-regulated senescence and inbred staygreen with the ability to partition excess sugar into alternative sink tissues such as stalks (Kumar et al. 2019). These traits have significant agronomic importance, and further work should be done to study how this relationship can be used to improve agronomic characteristics of maize. We recommend that future studies continue to investigate the relationship of these senescence mechanisms by measuring senescence of the hybrids past the black-layer formation to quantify end-of-season senescence in response to natural sink disruption.

Traits quantifying the rate of change for source-sink-related senescence did not generally show significant correlations with yield or ear photometry traits. The major exception was the rate of change in CCI for pollinated plants, which is equivalent to the rate of senescence for normal plants from 425 to 600 GDD after pollination. While these results are interesting, we did not focus on them in this paper because we are specifically interested in senescence induced by sink disruption, not senescence writ large.

If source-sink-regulated senescence was a strong driver of yield, we would expect it to have undergone convergent selection during the breeding process. Source-sink-regulated senescence was not strongly correlated with yield, and the senescence trait was widely distributed among exPVP germplasm, indicating that the senescence phenotype has not been a target of selection. Within the Iodent and Stiff Stalk families, significant and opposite correlations were observed. If source-sink senescence were a driver of yield for within-family genetic crosses, we would expect directional selection for yield during the era of open-pollinated varieties to have increased the strength of the senescence response in Iodents and decreased the strength of senescence response in Stiff Stalks. However, we observed the opposite—this phenotype is widely distributed in both families and that the Stiff Stalks senesce more strongly in response to sink disruption than the Iodent inbred lines. One possible explanation is that B73, the most prominent founder of the Stiff Stalk family, has an incredibly strong source-sink senescence response. Since commercial breeding programs select for yield of hybrids made from inter-family crosses and there is no correlation between yield of inter-family hybrids and senescence of the parents, the senescent alleles from B73 could remain in the Stiff Stalk population without experiencing strong selection during the modern era of breeding even though those alleles seem to carry a yield penalty within the Stiff Stalk pool. A similar story could have played out within the Iodent family as well. One way to test this hypothesis would be to compare the distribution of source-sink senescence in early vs late exPVP inbred lines. As the inbred lines themselves are selected for higher yield, the senescence phenotype should decrease within the Stiff Stalk population over time and increase within the Iodents.

### Loci linked to source-sink-regulated senescence

The GWAS analysis for senescence traits identified five candidate loci. Genes located in these regions provide a range of hypotheses for the mechanisms behind differential senescence response to sink disruption in maize and how this may be linked to kernel size and other yield traits. These genes are putative candidates, which require functional testing.

The SNP 1–24,655,033 association is near (3 Mbp) putative candidate genes identified in the network analysis of senescence genes in a previous study (Sekhon et al. 2019), near three high likelihood SNPs identified in a GWAS of SSRS in the Wisconsin diversity panel (Kumar et al. 2023), and located within a QTL hotspot identified in a metastudy of kernel trait loci (Wang et al. 2023). The 125 kb genomic region flanking SNP 1–24,655,033 contains four protein-coding genes. SRL5 maintains cuticular wax structure in maize, which helps with leaf integrity and drought tolerance (Pan et al. 2020). SFP1 is a sulfate permease involved in root uptake of sulfur from soil, and *sfp1* is abundantly expressed in maize roots and not leaves (Bolchi et al. 1999; Piłsyk and Paszewski 2009; Portwood et al. 2019). The F-box protein *gid4* encodes a soluble gibberellic acid (GA) receptor, whose loss of function leads to reduced plant stature (Ueguchi-Tanaka et al. 2005), and altered GA signaling is known to impact leaf senescence (Ritonga et al. 2023). GA receptors undergo proteasomal degradation after association with GA signaling repressor DELLA proteins in a GA-dependent manner (Eckardt 2007). Intriguingly, loss of function of DELLA proteins promoted premature leaf senescence in Arabidopsis, but loss of function of GA receptors delayed leaf senescence, potentially due to normal accumulation of DELLA proteins (Chen et al. 2014). Therefore, the role of GA biosynthesis and signaling in senescence needs to be explored in maize and the association of SNP 1–24,655,033 with *gid4* could be driven by allelic variation in our maize exPVP panel.

The 125 kb genomic region flanking SNP 3–115,601,313 contained three protein-coding genes, with only two expressed genes. The nearest expressed gene at ~ 86 kb from the top SNP encodes *calcineurin B-Like 2 (cbl2*; Zm00001d041392). In maize, the CBL gene family functions in calcium signaling, ABA signaling, and response to abiotic stress, which could influence the source-sink-regulated senescence response (Zhang et al. 2016). An Arabidopsis homolog of *cbl2* increases ATPase activity in response to calcium signal after alkalinity stress to maintain cellular homeostasis (Yang et al. 2019). Functional characterization is needed to determine if *clb2* functions similarly in maize.

The 125 kb genomic region around SNP 3–157,623,549 carried four protein-coding genes with the nearest gene at ~ 35 kb. Maize gene Zm00001d042257, ~ 57 kb from SNP 3–157,623,549, is an homolog of Arabidopsis gene (AT5G43750) that encodes photosynthetic NDH subunit of subcomplex B5 (PnsB5) and is targeted to the chloroplast (Ifuku et al. 2011). The constitutively expressed PnsB5 in Arabidopsis enhanced oxidative stress induced by t-butyl hydroperoxide and herbicide paraquat (Luhua et al. 2008). The *zmpnsb5* gene is abundantly expressed in mature leaves in maize (MaizeGDB) and could be involved in the response to oxidative stress caused by excess sugar accumulation in non-pollinated maize plants (Kumar et al. 2023).

The genomic region around SNP 4–241,458,461 contained 11 protein-coding genes, with multiple genes that could regulate senescence. The nearest expressed gene to this SNP at ~ 5 kbp with a known function is Zm00001d053807, which encodes *camouflage2* (*cf2*) (Huang et al. 2009). Its paralog *camouflage1* (*cf1*) encodes chloroplast-localized porphobilinogen deaminase enzyme in porphyrin biosynthesis pathway that is abundantly expressed in leaves (Huang et al. 2009). The loss-of-function alleles of *cf1* cause bundle sheath cell death and produce non-clonal sectors (Huang et al 2009). Loss of porphobilinogen deaminase activity reduces downstream heme biosynthesis, a cofactor critical for catalase activity (Labbe-Bois et al. 1977), and results in increased reactive oxygen species (ROS) leading to bundle sheath cell death (Huang et al. 2009). It is likely that high ROS scavenging is required during source-sink-regulated senescence, as proposed by a previous study in maize (Kumar et al. 2023), and natural variation at *cf2* in porphyrin biosynthesis can affect ROS scavenging activity via the heme pathway. Another gene linked to this locus (~ 54 kbp from SNP 4–241,458,461) is *defective kernel 10* (*dek10;* Zm00001d053802) that encodes a PPR protein localized in mitochondria, and its loss of function results in small kernels, miniature seedlings, and delayed growth in maize (Qi et al. 2017). *Dek10* was also previously proposed as a putative candidate gene in a maize senescence gene network analysis (Sekhon et al. 2019), and a GWAS in the Wisconsin diversity panel identified a candidate SNP linked to this locus (Kumar et al. 2023). However, no senescence phenotype was reported in a study of *dek10* loss-of-function maize alleles (Qi et al. 2017). Another gene at this locus, Zm00001d053812, encodes *glycerophosphodiester phosphodiesterase4* (*gpx4*) whose loss of function in maize compromises the remobilization of phosphorus from senescing leaves to young leaves, resulting in a staygreen phenotype under phosphate starvation (Wang et al. 2021). It is likely that the variation in *gpx4* could influence the rate of senescence in response to sink disruption.

The fifth SNP association at 6–142,491,809 was unique as it was not associated with any senescence phenotypes measured at a single timepoint but was associated with rate of senescence measured by different traits (Rate_NonPol, Rate_NonPoladj, Rate_Difference, and Rate_SSRS) across 425 and 600 GDD post-anthesis. None of the four protein-coding genes linked to this SNP have been previously linked to natural senescence or source-sink-regulated senescence (40S ribosomal protein S5-2, biotin synthase, transducin/WD40 repeat-like superfamily protein, and UDP-glucuronic acid decarboxylase 4 related).

Interestingly, the SNPs identified by our work on chromosomes 1 and 4 are within kernel size hotspots identified by a recent metastudy of kernel size-related QTL (Wang et al. 2023). This could provide an explanation for the correlation we observed of source-sink-regulated senescence with kernel size and yield, either through genetic linkage or shared gene action. However, the five Bonferroni SNPs associated with senescence traits in our study were not directly correlated with hybrid yield, senescence, and ear photometry traits at a significant level and an enrichment analysis did not find evidence for overlap between the source-sink-regulated senescence loci in inbreds and loci associated with hybrid yield, senescence, and ear photometry in hybrids.

### Test of GWAS candidates using a mechanistic framework

Unlike Arabidopsis, maize has limited reverse genetic resources, but decades of careful mutant studies have produced a set of well-characterized mutants that underpin specific molecular processes. We therefore sought to test GWAS candidates by anchoring mechanisms of senescence regulation not to the specific candidate genes identified by GWAS, but to the broader biochemical pathways that these genes represent. The two pathways identified in the GWAS study, gibberellic acid signaling (*d8* and *d9*) and porphyrin biosynthesis (*cf1*), have been previously well-characterized using maize mutants (Best and Dilkes 2022; Huang et al. 2009).

DELLA repression of the GA signaling pathway has been shown to delay leaf senescence in Arabidopsis (Chen et al. 2014), and staygreen in maize is positively correlated with delayed source-sink-regulated senescence (Kumar et al. 2019). Consistent with these results, the mutant F1 hybrid siblings for both *d8* and *d9* were observed to accumulate higher initial chlorophyll in all genetic backgrounds compared to wild-type F1 hybrid siblings (Fig. 8). However, both mutant and wild-type siblings displayed a strong senescence response to non-pollination, and *D8-Mpl* and *D9-1* mutants lost more chlorophyll in absolute terms than their wild-type siblings by 425 GDD after anthesis. By 600 GDD after anthesis, both mutant and wild-type siblings had lost all chlorophyll in the non-pollinated treatments. It seems that observed differences in *D8-Mpl* and *D9-1* mutants that carry dominant-negative alleles were due to an elevation in initial chlorophyll levels rather than an attenuation of the senescence response to sink disruption. It is possible that the association at 1–24,655,033 was driven by *gid4* attenuating the senescence response to non-pollination via GA signaling pathways.

The porphyrin pathway was proposed based on the association at SNP 4–241,458,461 that was linked to a paralog of *cf1*. The enzymes in ROS scavenging pathway have been previously proposed to regulate SSRS in maize (Kumar et al. 2023). We expected that a block in porphyrin biosynthesis in *cf1* mutants would exacerbate ROS and enhance senescence under both pollinated and non-pollinated conditions. However, no negative effect of *cf1* mutation on the chlorophyll levels was observed under the pollinated treatment, even more surprising, slightly higher chlorophyll levels were observed under non-pollinated conditions (Fig. 8). In light of this genetic test, we caution the broader field from interpreting the role ROS might play in source-sink-regulated senescence, particularly as it relates to downstream impacts of porphyrin pathway. These results do not disprove the ROS hypothesis, but fail to support it. Future mutant studies should test other enzymes further downstream in the heme pathway to better understand the role of ROS scavenging in senescence.

Future work with mutants, fine-mapping, and functional gene analysis will allow us to uncover the mechanisms driving source-sink-regulated senescence and the fundamental processes of senescence. Given the limited reverse genetic resources in maize, we propose that SNP–trait associations can be assigned to biochemical pathways or molecular processes using candidate genes in the genomic intervals, and the impact of those molecular processes on the phenotype can be explored using existing mutant resources for broader genetic understanding of a phenotype.

## Conclusion

Source-sink-regulated senescence of maize inbred lines was correlated with increased kernel numbers and yield of intra-family hybrids but was not generally correlated with yield of hybrids made from crosses between different heterotic groups. This is consistent with the wide distribution of the source-sink senescence phenotypes across diverse genetic groups. The mechanisms behind source-sink-regulated senescence may contribute to increased evolutionary fitness, potentially through increased progeny numbers, but are not strong drivers of yield in a commercial breeding context. This allows the genetic factors controlling the trait to remain widely distributed in the elite germplasm pool as an evolutionary artifact. The presence of multiple Bonferroni SNP in genomic regions associated with kernel traits could explain the observed correlation of source-sink-regulated senescence with kernel size and yield, either through genetic linkage or shared gene action. Single factor testing of DELLA mutants supports the conclusion that the GA-DELLA signaling pathway may attenuate the senescence response to sink disruption in maize. Future work with mutants, fine-mapping, and functional gene analysis will be required to uncover the mechanisms of gene action. Induction of senescence through sink disruption is a cost-effective and reliable phenotype, which can be used to map genes associated with fundamental processes of senescence.

## Supplementary Information

Below is the link to the electronic supplementary material.

Supplementary file1 (PPTX 9128 kb)

Supplementary file2 (XLSX 26 kb)
