# Supplementary material for: Yield impact of source-sink-regulated senescence in hybrid maize and genetic architecture in exPVP inbreds
Source: Theor Appl Genet. 2026 Apr 27;139(5):135. doi: 10.1007/s00122-026-05210-z (PMC13121282; doi:10.1007/s00122-026-05210-z)
Supplement: Supplementary file 1 — Supplementary file1 (PPTX 9128 kb) [file 122_2026_5210_MOESM1_ESM.pptx]

## Slide 1
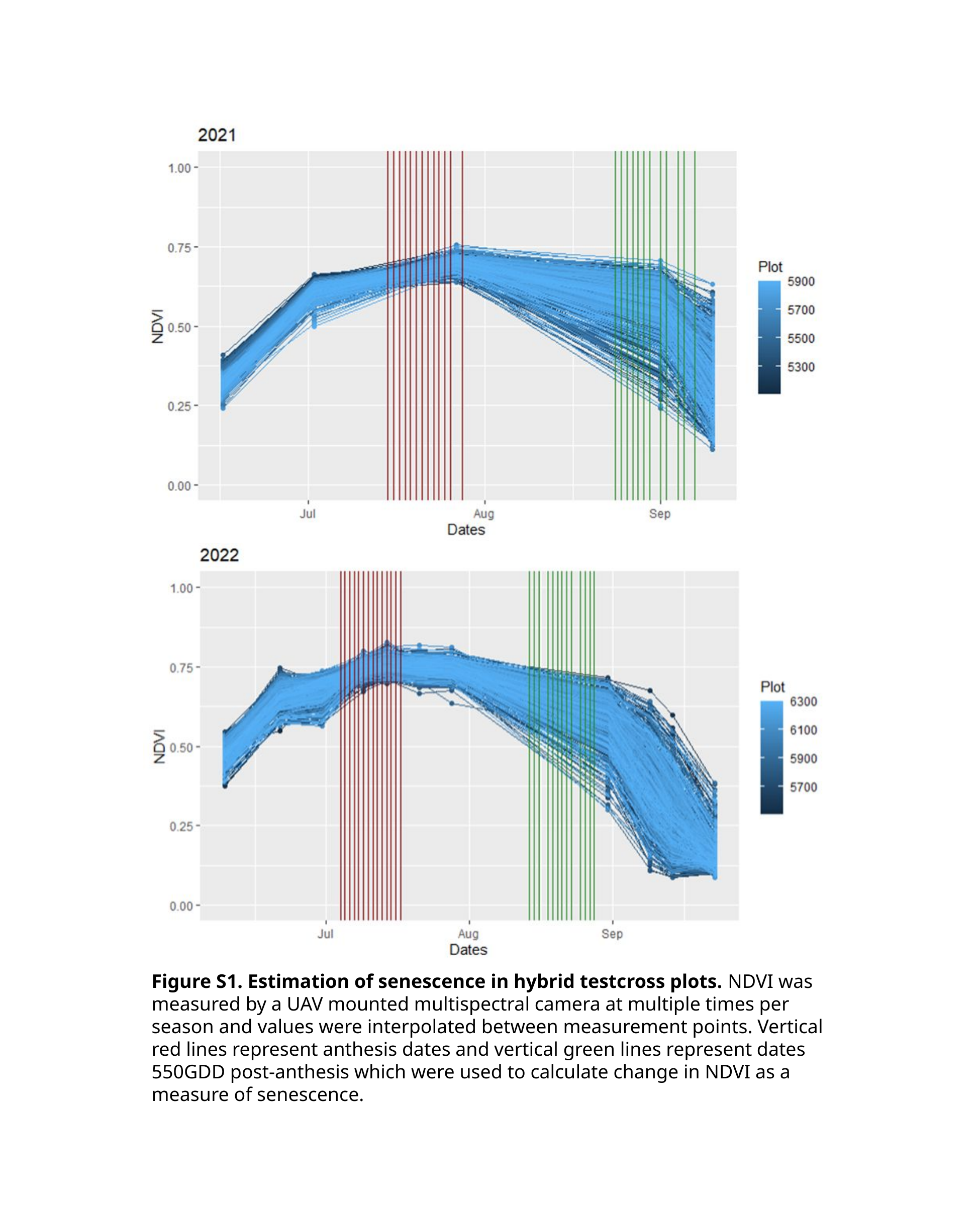

Figure S1. Estimation of senescence in hybrid testcross plots. NDVI was measured by a UAV mounted multispectral camera at multiple times per season and values were interpolated between measurement points. Vertical red lines represent anthesis dates and vertical green lines represent dates 550GDD post-anthesis which were used to calculate change in NDVI as a measure of senescence.

## Slide 2
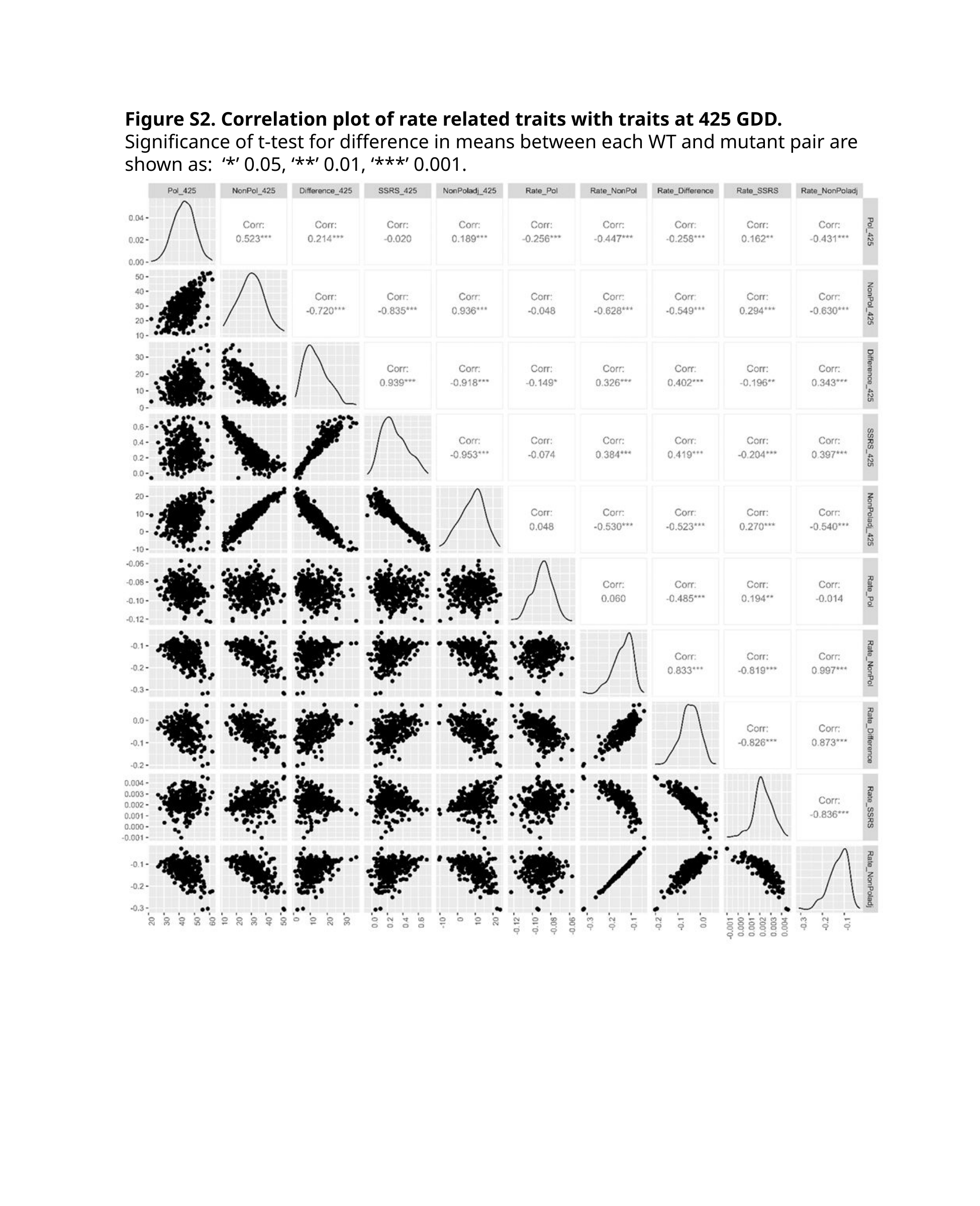

Figure S2. Correlation plot of rate related traits with traits at 425 GDD. Significance of t-test for difference in means between each WT and mutant pair are shown as:  ‘*’ 0.05, ‘**’ 0.01, ‘***’ 0.001.

## Slide 3
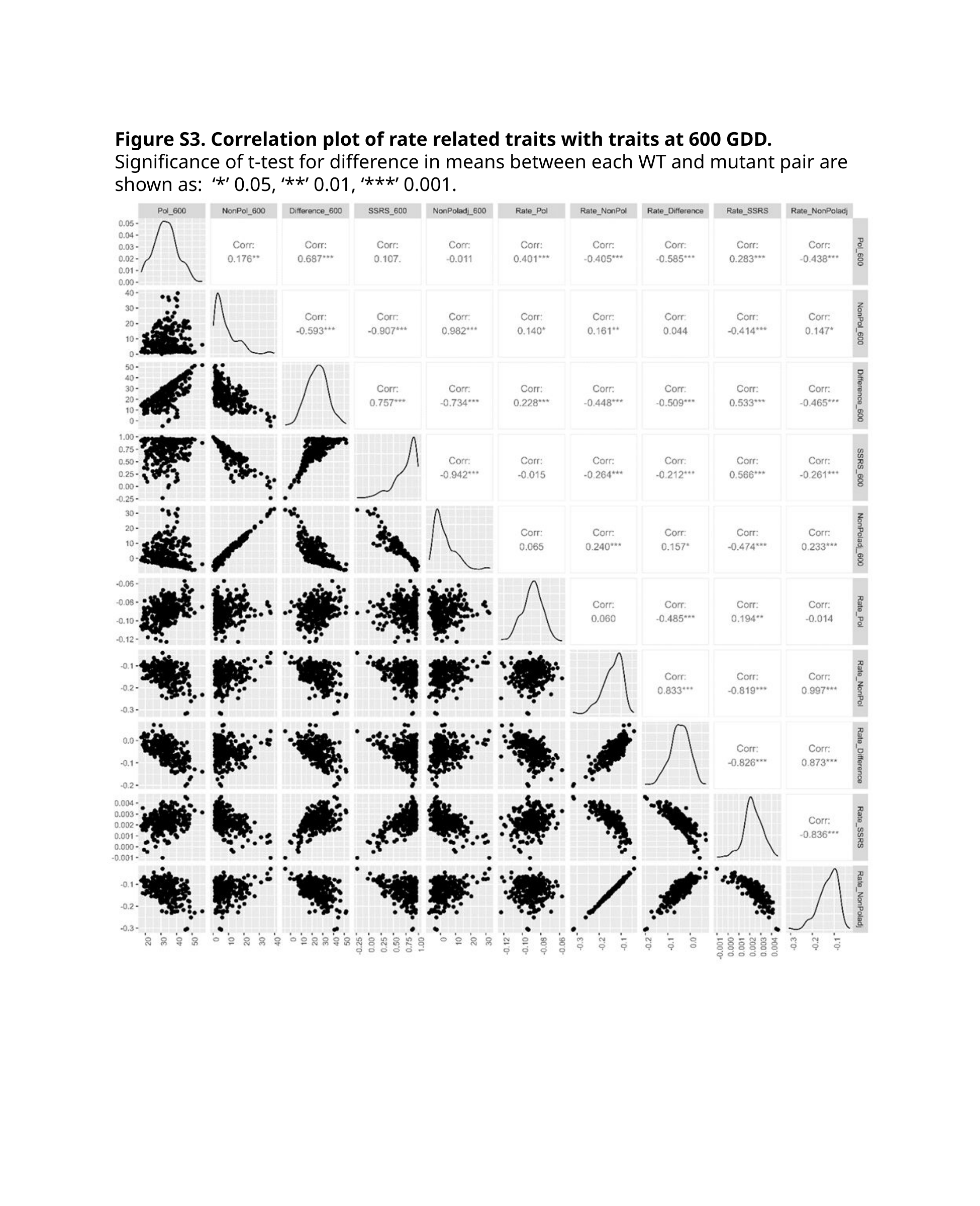

Figure S3. Correlation plot of rate related traits with traits at 600 GDD. Significance of t-test for difference in means between each WT and mutant pair are shown as:  ‘*’ 0.05, ‘**’ 0.01, ‘***’ 0.001.

## Slide 4
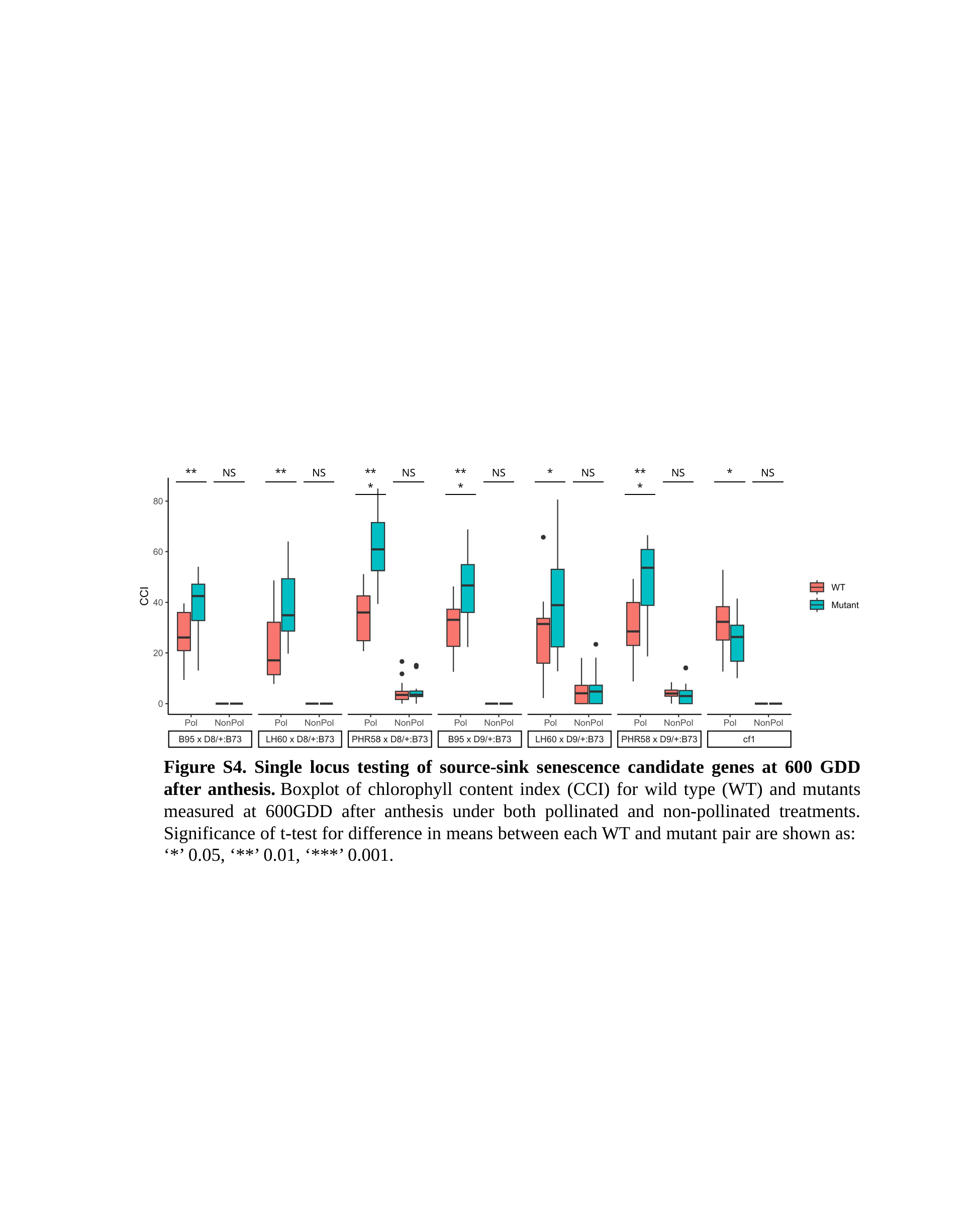

| \*\* |
| --- |
| NS |
| --- |
| \*\* |
| --- |
| NS |
| --- |
| \*\*\* |
| --- |
| NS |
| --- |
| \*\*\* |
| --- |
| NS |
| --- |
| \* |
| --- |
| NS |
| --- |
| \*\*\* |
| --- |
| NS |
| --- |
| \* |
| --- |
| NS |
| --- |
Figure S4. Single locus testing of source-sink senescence candidate genes at 600 GDD after anthesis. Boxplot of chlorophyll content index (CCI) for wild type (WT) and mutants measured at 600GDD after anthesis under both pollinated and non-pollinated treatments. Significance of t-test for difference in means between each WT and mutant pair are shown as: ‘*’ 0.05, ‘**’ 0.01, ‘***’ 0.001.

## Slide 5
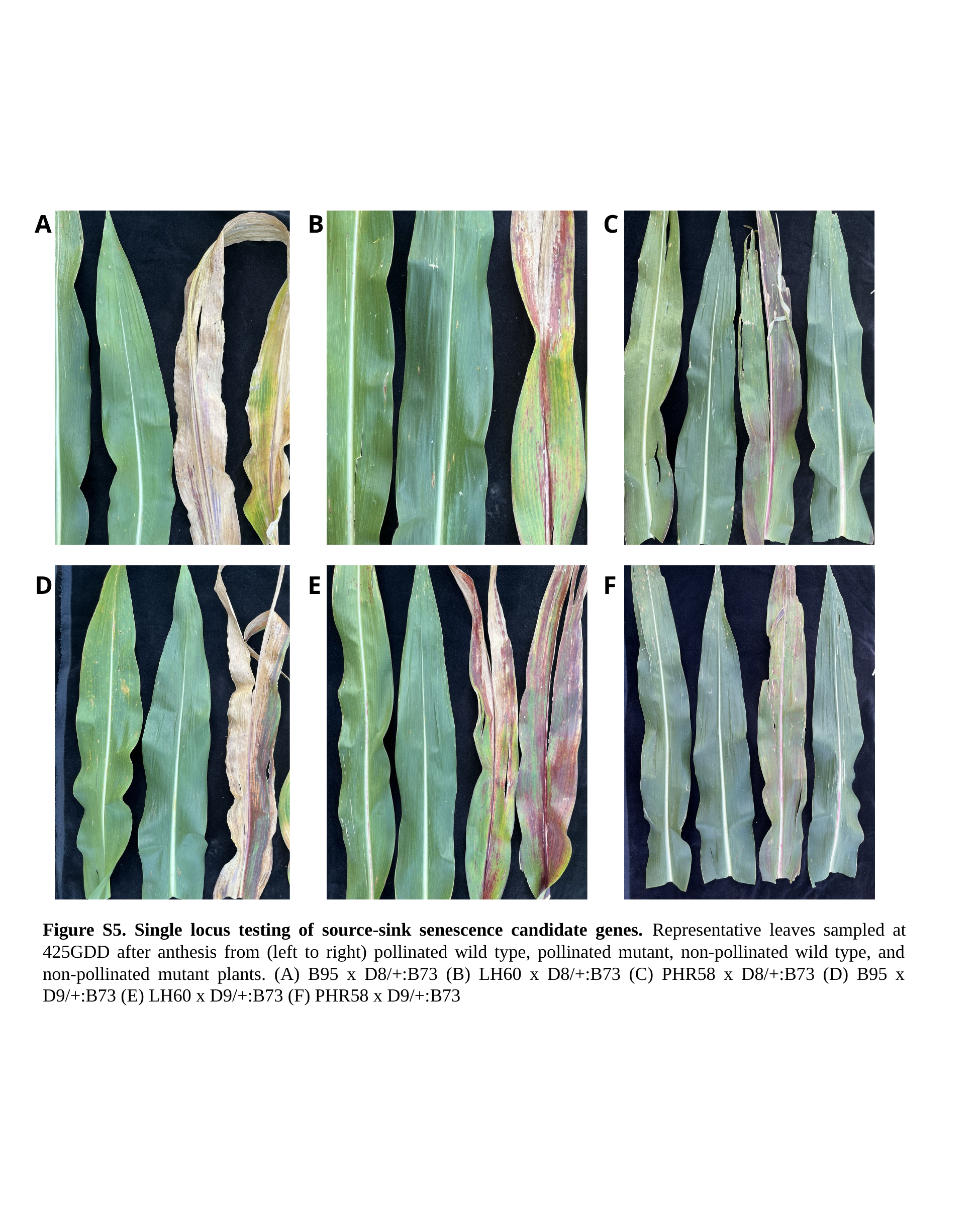

A
B
C
D
E
F
Figure S5. Single locus testing of source-sink senescence candidate genes. Representative leaves sampled at 425GDD after anthesis from (left to right) pollinated wild type, pollinated mutant, non-pollinated wild type, and non-pollinated mutant plants. (A) B95 x D8/+:B73 (B) LH60 x D8/+:B73 (C) PHR58 x D8/+:B73 (D) B95 x D9/+:B73 (E) LH60 x D9/+:B73 (F) PHR58 x D9/+:B73
